# A Guide to Targeting the Endocannabinoid System in Drug Design

**DOI:** 10.3390/ijms21082778

**Published:** 2020-04-16

**Authors:** Adam Stasiulewicz, Katarzyna Znajdek, Monika Grudzień, Tomasz Pawiński, Joanna I. Sulkowska

**Affiliations:** 1 Department of Drug Chemistry, Faculty of Pharmacy, Medical University of Warsaw, Banacha 1, 02-097 Warsaw, Poland; monika.grudzien@wum.edu.pl (M.G.); tomasz.pawinski@wum.edu.pl (T.P.); 2 Interdisciplinary Laboratory of Biological Systems Modelling, Centre of New Technologies, University of Warsaw, Banacha 2c, 02-097 Warsaw, Poland; k.znajdek@cent.uw.edu.pl; 3 Faculty of Pharmacy, Medical University of Warsaw, Banacha 1, 02-097 Warsaw, Poland; 4 Faculty of Chemistry, University of Warsaw, Pasteura 1, 02-093 Warsaw, Poland; 5 Materials and Process Simulation Center, California Institute of Technology, Pasadena, CA 91125, USA

**Keywords:** endocannabinoid system, ECS, CB1, CB2, FAAH, MAGL, molecular target

## Abstract

The endocannabinoid system (ECS) is one of the most crucial systems in the human organism, exhibiting multi-purpose regulatory character. It is engaged in a vast array of physiological processes, including nociception, mood regulation, cognitive functions, neurogenesis and neuroprotection, appetite, lipid metabolism, as well as cell growth and proliferation. Thus, ECS proteins, including cannabinoid receptors and their endogenous ligands’ synthesizing and degrading enzymes, are promising therapeutic targets. Their modulation has been employed in or extensively studied as a treatment of multiple diseases. However, due to a complex nature of ECS and its crosstalk with other biological systems, the development of novel drugs turned out to be a challenging task. In this review, we summarize potential therapeutic applications for ECS-targeting drugs, especially focusing on promising synthetic compounds and preclinical studies. We put emphasis on modulation of specific proteins of ECS in different pathophysiological areas. In addition, we stress possible difficulties and risks and highlight proposed solutions. By presenting this review, we point out information pivotal in the spotlight of ECS-targeting drug design, as well as provide an overview of the current state of knowledge on ECS-related pharmacodynamics and show possible directions for needed research.

## 1. Introduction

*Cannabis sativa* has been used for medical and recreational purposes for millennia [1]. The attention this plant has been a subject of resulted in the exploration of the way it influences the human organism. This opened a new chapter in modern pharmacology. The isolation of main constituents of *Cannabis*, Δ9-tetrahydrocannabinol (Δ9-THC) and cannabidiol (CBD) in the 1960s [2] led to the latter discovery of cannabinoid receptors (CB receptors or CBRs) [3,4,5] and to the identification of their endogenous ligands [6,7]. This initiated wide research on this biological system, called the endocannabinoid system (ECS). Today, we know that ECS comprises cannabinoid receptors type 1 and 2 (CB1 and CB2), their endogenous ligands—endocannabinoids (eCBs), enzymes, transporters, and other receptors. In this review, we will show the way in which the proteins of ECS may be possibly targeted in the treatment of various diseases and disorders. We will highlight recent, prominent studies, the most promising strategies, and the most interesting directions one could follow in ECS-related drug design.

**Figure 1:**
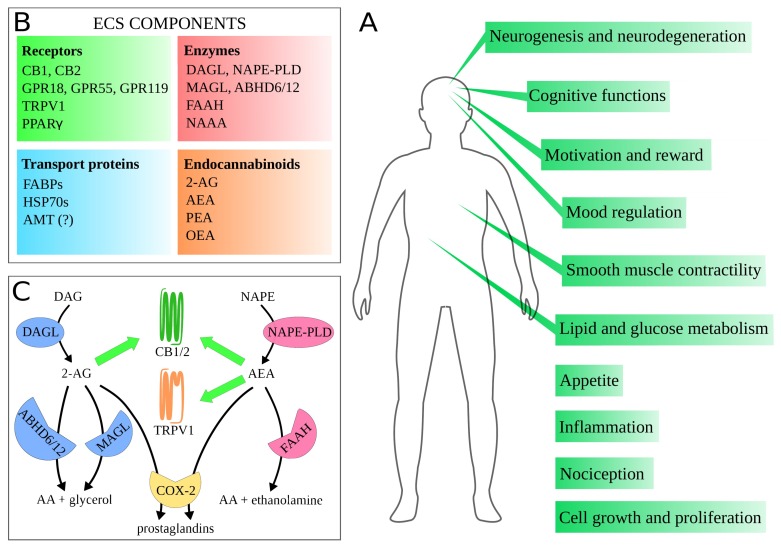
(**A**) physiological processes and functions that the endocannabinoid system (ECS) takes part in. In this figure, we gathered probably the most crucial ones in terms of potential therapeutic applications, described in detail in this review; (**B**) components of the ECS; (**C**) simplified biochemical pathways of the two main endocannabinoids: 2-arachidonoylglycerol (2-AG) and N-arachidonoylethanolamine, also called anandamide (AEA). Blue shapes depict enzymes associated mainly with 2-AG, pink ones—with AEA. Green arrows indicate activation of specific receptors by the endocannabinoids. Abbreviations: 2-AG, 2-arachidonoylglycerol; AA, arachidonic acid; ABHD6/12, α/β hydrolase domain 6 or 12; AEA, N-arachidonoylethanolamine (anandamide); AMT, anandamide membrane transporter; CB1/2, cannabinoid receptor type 1 or 2; COX-2, cyclooxygenase 2; DAG, diacylglycerol; DAGL, diacylglycerol lipase; FAAH, fatty acid amide hydrolase; FABPs, fatty-acid-binding proteins; GPR18/55/119, G protein-coupled receptor 18 or 55 or 119; HSP70s, 70 kilodalton heat shock proteins; MAGL, monoacylglycerol lipase; NAAA, N-acylethanolamine acid amidase; NAPE, N-acylphosphatidylethanolamine; NAPE-PLD, N-acylphosphatidylethanolamine-hydrolyzing phospholipase D; OEA, oleoylethanolamine; PEA, palmitoylethanolamide; PPARγ, peroxisome proliferator-activated receptor γ; TRPV1, transient receptor potential vanilloid type 1 channel.

The discovery of cannabinoid receptors and endocannabinoids began the extensive research on this newly found physiological system. The considerable expansion of knowledge on ECS during the last few decades resulted in a paradigm shift. ECS, despite being named after a psychoactive plant, is in fact a very important system in the human organism. CB1 is among the most abundant receptors in the central nervous system (CNS), and is a major regulatory factor for multiple neurons, including glutaminergic, GABAergic, dopaminergic, etc. [8]. Additionally, CB1 is present in many important peripheral organs and tissues, while CB2 is most notably in immune cells [9]. Thus, ECS takes part in a vast array of physiological processes and functions, including: mood regulation, pain management, cognitive functions, reward, appetite, fat and glucose metabolism, neurogenesis and neuroprotection, inflammation and immune functions, smooth muscle contractility, cell proliferation, and many others (Figure 1A). Thus, ECS has been viewed as a potential therapeutic target in multiple diseases and disorders. Compounds aiming proteins of ECS have been used or studied as a treatment of pain, seizures, psychiatric disorders, obesity, metabolic diseases, neurodegenerative diseases, and cancer. Alas, to date, these attempts have not achieved the desired success.

ECS is a complex system that has major implications for the safety of pharmacotherapy. The multi-directional character of this system poses a threat of adverse effects. The most notorious example is rimonabant—a CB1 inverse agonist used as an anti-obesity agent. Drugs containing rimonabant were approved in Europe in 2006. Alas, they exhibited major psychiatric adverse effects, including anxiety and depression, and in some cases led to consequent suicides. Those drugs were withdrawn in 2008 [10]. On the superficial level, the idea was logical: decreasing CB1 signaling reduces appetite and fat accumulation. Unfortunately, a multi-purpose nature of ECS was neglected. Downregulation of CB1 signaling is known to negatively alter mood and emotional state, and this knowledge was available even before the approval of the drugs containing rimonabant [11]. Thus, when designing drugs targeting ECS components, one has to bear in mind the possible multi-directional effects, which may be beneficial or deleterious depending on the specific ECS-targeting drug. In addition, downregulation of endocannabinoid signaling has to be planned with caution, as ECS is crucial for regulating many physiological functions.

Analogically, activation of cannabinoid receptors may be a powerful therapeutic strategy. However, it also comes with several obstacles which may be illustrated with the instance of *Cannabis sativa*. This plant is recently going through a renaissance, with a considerable increase in its medical use. It seems to be a valuable alternative for some drugs. Unfortunately, the therapeutic profile of *Cannabis* is not perfect. Although the administration of this medicinal plant’s preparations is generally well tolerated, in some cases, it may lead to possible risks of addiction, various adverse effects, and cognitive dysfunctions due to a long-term therapy [12]. Additionally, the efficacy of the use of *Cannabis* in several indications is constantly discussed. For example, the results of clinical trials regarding therapy of chronic pain are inconclusive [13,14]. Though *Cannabis sativa* seems to be a promising direction, cannabinoids with a potentially better pharmacological profile have been sought in other plants in recent years. For example, CB1 ligands were found in *Daucus carota*, *Piper methysticum*, and *Heliopsis helianthoides*. However, these phytocannabinoids have low to moderate affinities towards CB1, in some cases unknown intrinsic activities, and in most instances clinical applicability yet to be determined [15]. Thus, more research is needed in this area, and so far *Cannabis* holds its reign in the field. Nevertheless, the problems this plant faces show the importance of considering typical adverse effects caused by CB1 activation, and highlight the necessity of avoiding them in modern ECS-related drug design.

Because of numerous problems that ECS targeting creates, which we illustrated in the above examples, this system is sometimes referred to as “undruggable”. This concept was proved to be entirely wrong by some drugs known for a long time. The best example would be paracetamol (acetaminophen)—one of the most often used drugs all over the world. It was initially believed to act similarly to nonsteroidal anti-inflammatory drugs (NSAIDs) and was studied for its potential impact on the prostanoid system [16,17,18]. Today, we know that it has a complex mechanism of action (MOA) and acts via cyclooxygenase 3 (COX-3) inhibition [19], serotonin receptor 3 (5-HT3) antagonism [20,21], nitric oxide synthase (NOS) inhibition [22], and CB1 agonism [23]. In 2006, Ottani et al. showed that the latter mechanism may be the most important one for paracetamol’s properties. They blocked CB1 with two antagonists—SR141716A and AM281. In both cases, paracetamol’s analgesic activity was prevented [23]. In 2017, Sharma et al. proved that paracetamol is metabolized in vivo to N-arachidonoylaminophenol (AM404) [24], which is the anandamide reuptake inhibitor [25], as well as weak CB1 [26] and TRPV1 agonist [27]. Another analgesic, metamizole (dipyrone), was also found to act via ECS [28,29]. Fenofibrate, a peroxisome proliferator-activated receptor α (PPARα) agonist, was proved to be a CB1 and CB2 agonist and at higher concentrations a CB1 negative allosteric modulator (NAM) [30].

The story of drugs acting via ECS does not end at the known drugs with unknown endocannabinoid MOA. The pursuit of novel ECS-targeting compounds continues. The involvement of ECS in multiple physiological processes provides an immense therapeutic potential that remains hard to be given up. Additionally, recent elucidation of CB1 [31] and CB2 [32] tertiary structures creates a new stimulus for the design of novel ligands for these two important receptors. Moreover, the knowledge on ECS physiology is constantly growing, allowing us to better understand chances and risks in targeting this system. It is worth mentioning that endocannabinoids also play a role in the pathophysiology of various diseases and disorders, thus making the ECS even more desired target. As in this review, we focus on the proteins of ECS; the readers are directed to other recent reviews which focus on eCBs’ biochemistry [33] and their impact on specific pathological conditions [34]. Because of all these factors, there have been numerous attempts and proposed solutions to overcome aforementioned difficulties. The best known of these proposals include: (1) development of peripheral CB1 agonists that do not cross the blood–brain barrier, and therefore do not cause typical phytocannabinoid-like adverse effects; (2) using CB1 neutral antagonists instead of inverse agonists or CB1 peripheral antagonists/inverse agonists to avoid rimonabant-like adverse effects. These are sometimes called the “second generation” of synthetic cannabinoids [35]. In this review, we cover and highlight these and many more proposals for possibly effective and safe ECS targeting, with the division for pathophysiological areas, in which drugs could potentially be used. We also stress possible benefits and risks, and limitations of the state of knowledge on the involvement of specific molecular targets in potential applications. The data gathered in this review may be treated as a signpost. It enables the rational selection of a molecular target for drug design. In addition, it gathers information on the involvement of ECS in various physiological functions and diseases in one place and shows possible directions for much needed research.

## 2. Endocannabinoid System

ECS comprises cannabinoid receptors type 1 and 2 (CB1 and CB2), their endogenous ligands—endocannabinoids (eCBs), and enzymes responsible for eCBs’ synthesis and degradation (Figure 1B). eCBs include two main ones: 2-arachidonoylglycerol (2-AG) and N-arachidonoylethanolamine, better known as anandamide (AEA), but also palmitoylethanolamide (PEA), oleoylethanolamine (OEA), and others [36]. Simplifying, 2-AG is synthesized by diacylglycerol lipase (DAGL) and degraded by monoacylglycerol lipase (MAGL or MGL) and α/β hydrolase domain 6 and 12 (ABHD6 and ABHD12) [37]. AEA is synthesized by N-acylphosphatidylethanolamine-hydrolyzing phospholipase D (NAPE-PLD) and degraded by fatty acid amide hydrolase (FAAH) [38] (Figure 1C). Additionally, there are other proteins involved in ECS. AEA is transported by fatty-acid-binding proteins (FABPs), 70 kilodalton heat shock proteins (HSP70s), and probably anandamide membrane transporter (AMT), although AMT’s existence has not been directly proven [38]. Apart from CB1 and CB2, eCBs also bind to other proteins, most notably to G protein-coupled receptor (GPCR) 18 (GPR18) [39], GPR55 [40], GPR119 [41], transient receptor potential vanilloid type 1 (TRPV1) channel [42], and peroxisome proliferator-activated receptor γ (PPARγ) [43]. As some of these are parts of other physiological systems [44], it is hard to define the borders of ECS. Therefore, we will focus on core proteins of ECS, but will also cover others when it is necessary.

## 3. Molecular Mechanisms of the Main Proteins of ECS—Implications for Drug Design

Targeting the specific proteins of ECS also requires knowledge of basic concepts and nuances in their pharmacology at a molecular level. As this is a very broad topic, and there are many research articles and reviews regarding this area [45,46,47,48], we will limit this section to information that is crucial for understanding consecutive parts and will highlight the concepts vital for modern ECS-related drug design.

The main proteins of ECS, CB1 and CB2 are G protein-coupled receptors. As such, their ligands are characterized with both binding affinity and intrinsic activity. It is important that CBRs exhibit constitutive activity [49,50], and thus their ligands’ intrinsic activities vary from agonist, through partial agonist and antagonist, to inverse agonist. As up and downregulation of ECS-mediated signaling are desired in specific pathophysiological conditions, CBRs agonists and antagosists/inverse agonists may act as drugs. In the case of CB1, the distinction between antagonists and inverse agonists is especially important because of the psychiatric adverse effects of the latter. It is believed that these difficulties may be overcome by utilization of CB1 antagonists [51]. Antagonists are sometimes referred to as “neutral” in order to emphasize that a compound is an antagonist, not an inverse agonist.

CBRs are coupled mainly to G_i/o_ α subunits (Gα_i/o_). Upon agonist binding, or constitutively, the receptor’s conformation changes from inactive to active. This allows for exchanging guanosine diphosphate (GDP) for guanosine triphosphate (GTP) on the α subunit of the G protein. Then, Gα dissociates from Gβγ dimer and from the CBR. Gα inhibits adenylyl cyclase (AC) and subsequently the cyclic adenosine monophosphate (cAMP)-dependent pathway. Gβγ regulates mitogen activated protein kinases (MAPKs). Additionally, Gβγ of CB1 affects calcium and potassium channels [52]. However, CBRs bind also to other G protein types and to non-G proteins, most notably β-arrestin, and thus CBRs’ activation may lead to the onset of various signaling pathways [53]. The occurrence and ratios of different messenger proteins depend heavily on their expression which is tissue- or cell-specific [53,54]. Thus, it is important to explore the possibilities of designing site-specific CBRs agonists and/or delivery systems. Moreover, CBRs exhibit biased signaling, which means that structurally diverse agonists stabilize different ranges of active conformations of the receptors, and consequently lead to a preference for activation of different biochemical pathways [53]. Development of CB1 biased agonists may be possibly a valid strategy to avoid phytocannabinoid-like adverse effects [55].

Another possible way to design potential CBRs-targeting drugs with desired pharmacological profile is the utilization of the allostery. Both CB1 and CB2 comprise probably a few allosteric binding sites [56,57,58]. Allosteric modulators may have a few advantages in comparison to the orthosteric ligands. Allosteric binding sites in GPCRs are less conserved than orthosteric ones, thus simplifying the design of selective compounds [59]. Allosteric modulators act in the presence of the orthosteric ligands, including eCBs. Thus, allosteric modulators retain the spacial and temporal nature of the endocannabinoid signaling. This implicates several possible pharmacodynamical advantages, including: not exhibiting constant and long lasting activation or inhibition, saturability (ceiling effect), and potential for lesser CBR desensitization or downregulation. These features may result in reduction of the risks of adverse effects, behavioral tolerance, and overdosing [60,61,62,63]. Moreover, it was shown that CBRs’ allosteric modulators may also exhibit biased signaling [64,65].

Although CB1 and CB2 are the most extensively studied proteins of ECS, there are also important factors regarding other molecular targets. In the case of eCB degrading enzymes, MAGL and FAAH, it is important to distinguish between reversible and irreversible inhibition [66,67]. For MAGL, it was shown that its irreversible inhibitors, through prolonged inactivation of this enzyme, may cause physical dependence, CB1 desensitization, and impaired eCB-dependent synaptic plasticity [68]. Moreover, MAGL and FAAH form homodimers [66,69]. Recently, FAAH dimers have been proven to be dependent on the signaling between their subunits and its allosteric regulation. Thus, it may be possible to design allosteric modulators of FAAH [69]. TRPV1 is a non-selective channel. It may be activated by ions, capsaicin, and other chemical or physical stimuli. Some of these stimuli allosterically control the transition from close to open conformation of TRPV1, importantly through different allosteric paths and distinct conformational changes [70,71]. This characteristic may be utilized in order to design TRPV1 allosteric modulators with specific allosteric mechanisms, and thus lacking typical adverse effects [47].

## 4. Nervous System

### 4.1. Pain

Today, the endocannabinoid system is an obvious and well-established target for the therapy of pain. ECS is a part of an endogenous antinociceptive system. Activation of CBRs in peripheral, spinal, and supraspinal neurons suppresses nociceptive transmission. Moreover, CBRs are present in immune cells and regulate inflammatory responses [72,73]. Thus, CBRs’ ligands may be a valuable alternative or adjunctive to opioids [74]. However, the administration of drugs targeting the ECS, especially CB1, comes with a risk of cognitive adverse effects [75] or disruption of ECS regulation of reward system [76,77]. One of the strategies to avoid the aforementioned obstacles is to design ligands acting only on peripheral CB1. Recently developed 4-2-[-(1E)-1[(4-propylnaphthalen-1-yl)methylidene]-1H-inden-3-yl]ethylmorpholine (PrNMI) is an example of a peripherally restricted cannabinoid (PRCB). In rat model (0.25 mg/kg), it suppressed chemotherapy-induced peripheral neuropathy (CIPN) allodynia via CB1 agonism [78]. Another possible way to avoid CB1-related adverse effects is modulating CB1 allosterically which was achieved by GAT211—a new CB1 positive allosteric modulator (PAM). It showed efficacy in inflammatory and neuropathic pain models in mice (10–20 mg/kg). Additionally, it did not create physical dependence and its therapeutic efficacy, without tolerance, was maintained for a longer time than for CB1 orthosteric agonists or MAGL inhibitors [79].

There is also an increasing number of research on CB2 impact on nociception. Some evidence aims especially at neuropathic pain, for which CB2 on microglia and CNS-infiltrating macrophages plays an important role [80]. Recently, the first positive allosteric modulator of CB2 has been synthesized and exhibited antinociceptive activity (5–20 mg/kg) in a rodent neuropathic pain model [81].

In addition, other proteins of ECS are targeted in the fight against pain. The selective MAGL inhibitor, ABD-1970 (1–2 mg/kg), displayed antinociceptive and antipruritic activity in a rodent model, without causing typical cannabinoid adverse effects [82]. Selective FAAH inhibitors (10 mg/kg) were shown to be effective against neuropathic pain in mice [83]. However, the increase in AEA levels by FAAH inhibition leads not only to CB1 activation and consequent analgesic effect. Activation of presynaptic TRPV1 by AEA exhibits pro-nociceptive activity. Additionally, AEA is metabolized also to pro-inflammatory prostaglandins (PG) by COX. Therefore, dual FAAH/TRPV1 and FAAH/COX-2 inhibitors are proposed for the therapy of pain [84].

As ECS is an abundant system, it interacts and cooperates with other pharmacological systems and crosses other biochemical pathways. This is important in terms of mechanisms of action of several antinociceptive drugs. Ketamine, a non-competitive N-methyl-D-aspartate (NMDA) receptor antagonist, was found to act also via ECS. CB1 antagonist, AM251 (2-4 μg intracerebroventricularly), completely prevented ketamine’s (4 μg) central antinociception in rodent model. On the other hand, the inhibition of AEA reuptake by VDM11 (4 μg) increased antinociception caused by a low dose of ketamine (2 μg) [85]. Indomethacin morpholinamide (IMMA) (10 mg/kg), a novel COX-2 inhibitor, successfully reduced hyperalgesia and allodynia in the rodent model. CB2 antagonist, AM630 (3 mg/kg intraperitoneally), partially reversed IMMA’s action. This compound has a complex MOA, including reduction in prostaglandin E_2_ (PGE2) synthesis and CB2 activation [86]. Other studies focused on aripiprazole, an antypsychotic drug and a ligand of multiple dopamine (DA) and serotonin (5-HT) receptors. It was shown that aripiprazole (0.1–10 mg/kg intraperitoneally) exhibits antinociceptive action in a rodent model [87]. In mice, aripiprazole’s (0.1 mg) antinociceptive activity was prevented by both CB1 and CB2 antagonists. On the other hand, pain-relieving effects of aripiprazole were enhanced by co-administration of FAAH inhibitor, MAGL inhibitor, and AEA reuptake inhibitor [88]. Moreover, a case study on psychiatric patients showed that aripiprazole (2.5–15 mg/day) may exhibit antinociceptive action also in humans [89]. However, more research is needed in this area.

### 4.2. Seizures

**Figure 2:**
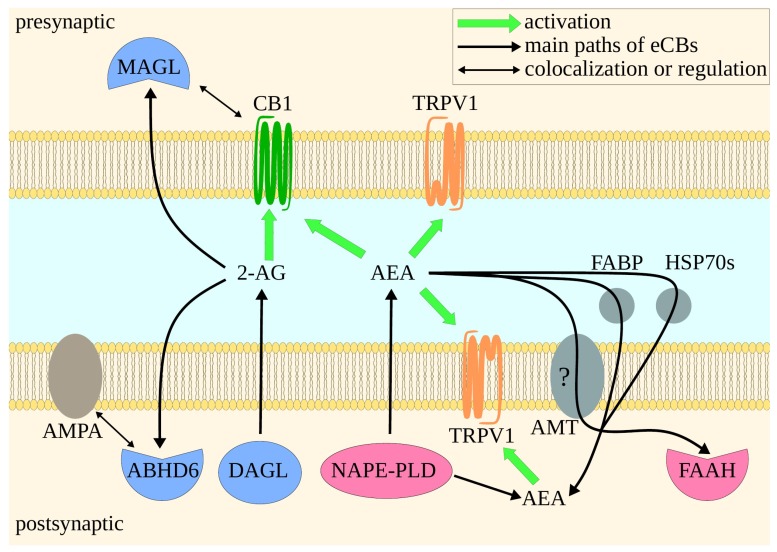
Simplified presentation of endocannabinoid signaling in the synaptic cleft. 2-AG is synthesized by DAGL in the postsynaptic neuron. After being excreted to the cleft, 2-AG activates presynaptic CB1. Finally, it is degraded by either MAGL in the postsynaptic neuron or by one of the ABHD’s in the presynaptic one. MAGL is colocalized with CB1. ABHD6 regulates AMPA. This is probably important for CB1-independent anti-seizure activity of ABHD6 inhibitors [92,95]. AEA is synthesized by NAPE-PLD. AEA may activate both CB1 and TRPV1, although the latter case is more complex. AEA may act on postsynaptic and presynaptic TRPV1. FABP, HSP70s, and probably AMT take part in AEA reuptake. Blocking this transport may be a valid therapeutic strategy, as it increases AEA extracellular levels, and thus leads to proportionally stronger activation of presynaptic TRPV1 than normal. FAAH inhibition, on the other hand, leads eventually to activation of TRPV1 in both synaptic neurons [93,94]. Green arrows indicate activation of receptors. Bold black arrows show main paths of 2-AG and AEA. Thin black arrows depict colocalization of CB1 and MAGL, and regulation of AMPA by ABHD6. Proteins are colored depending on their functions: enzymes associated mainly with 2-AG (blue), enzymes associated mainly with AEA (pink), eCB reuptake proteins (gray).

There is a constant debate on the possible use of *Cannabis sativa* and its constituents in seizures and epilepsy. Although this subject seems to be still open, it is a fact that ECS can impact aforementioned conditions [90]. CB1 agonists, such as WIN55,212, decreased seizure severity in rodent models. It has been shown that CB1 acts synergistically with 5-HT2B receptor, as co-administration of WIN55,212 (2 mg/kg, intraperitoneally) and 5-HT2B agonist RO60-0175 (3 mg/kg, intraperitoneally) decreased seizure severity and additionally reduced the incidence of seizures, while RO60-0175 (1–10 mg/kg, intraperitoneally) had no effect on its own [91]. The other possible direction is MAGL or FAAH inhibition. Although the increase in 2-AG and AEA levels should logically be connected with anti-seizure activity via CB1, recent studies on rodents indicate that only MAGL inhibition (e.g., JJKK048—1 mg/kg) results in anti-seizure activity, with 2-AG activating CB1 [92]. FAAH inhibition (e.g., URB597—80–160 μg intracerebroventricularly) leads to the increase in intracellular AEA levels, consequent TRPV1 activation and finally to pro-seizure activity [93,94]. On the other hand, anandamide reuptake inhibitor, LY21813240 (2.5 mg/kg), exhibited anti-seizure effects mediated by CB1 in the rodent model [92]. Therefore, it is important to distinguish between the increase in extracellular and intracellular levels of AEA. In addition, ABHD6 inhibitor, WWL70 (5 mg/kg), led to anti-seizure activity in the rodent model, though contrary to MAGL inhibition, in a CB1-independent manner [92]. It is possible that this mechanism is connected with the ABHD6 regulation of α-amino-3-hydroxy-5-methyl-4-isoxazolepropionic acid (AMPA) receptor [95]. Synaptic components of ECS are depicted in Figure 2.

Paracetamol is an analgesic and antipyretic drug. Interestingly, it was shown to exhibit anticonvulsant activity in vitro [96] and in rodent models (150–450 mg/kg intraperitoneally). It was hypothesized that this activity may be caused by AM404, an active metabolite of paracetamol, known to inhibit AEA reuptake, and activate TRPV1 and CB1 [97]. A later study proved that paracetamol’s anti-seizure activity in mice (300–450 mg/kg intraperitoneally) is CB1-independent, as AM251 (CB1 inverse agonist) did not prevent this activity. Additionally, TRPV1 antagonists reduced paracetamol anticonvulsant activity. All this information suggests that in rodents paracetamol displays anti-seizure properties via indirect TRPV1 agonism (through AM404), although more research is needed [98]. The contrary results showing that both TRPV1 agonism and antagonism may exhibit anti-seizure activity could be caused by influencing TRPV1 in different parts of the brain or TRPV1 in presynaptic or postsynaptic neurons. Moreover, it is important to state that paracetamol does not exhibit anticonvulsant activity in humans. When targeting ECS in coping with epilepsy and seizures, it is crucial to note considerable differences in the effects of ECS modulation in different species [99].

In the area of seizures, the greatest success was achieved by CBD. Epidiolex, an oral solution containing CBD, is an approved drug, with indications for two forms of epilepsy: Lennox–Gastaut syndrome and Dravet syndrome [100] (see: Section 11. Approved drugs and clinical trials). CBD has a complex mechanism of action, including CB1 negative allosteric modulation, TRPV1 agonism, and GPR55 antagonism. Moreover, it is a 5-HT1A agonist and interacts with multiple other pharmacologically relevant proteins [40,101,102,103]. The exact mechanism of CBD anticonvulsant activity is not entirely clear [104,105]. However, there have been multiple studies regarding this matter and CBD probably exhibits its anti-seizure properties not by the impact on CB1 or TRPV1 but rather via GPR55, 5-HT1A, and other molecular targets [104]. Thus, it may be a valid direction to study GPR55 antagonists as potential antiseizure drugs.

### 4.3. Anxiety

ECS is known to be a vital system for emotional processing and mood control. It takes part in regulation of stress and anxiety [106]. This can be a major problem in terms of adverse effects of new synthetic compounds acting via ECS, as shown by rimonabant [10]. On the other hand, targeting ECS may be a valid strategy to design novel psychotropic drugs, including anxiolytics.

Rimonabant, a potent CB1 inverse agonist, was an active ingredient of approved drugs used as anti-obesity agents. Due to rimonabant’s major psychiatric adverse effects, including depression and anxiety [10], those drugs were withdrawn. Additionally, several studies reported a link between downregulation of ECS with anxiety-like behaviors even before, also in the case of rimonabant itself [11,107]. Logically, CB1 agonists were believed to be a promising direction for developing a new class of anxiolytic drugs. Although cannabinoids are generally well tolerated, direct CB1 agonism in some cases may come with a risk of other psychotropic adverse effects [108,109,110]. Therefore, other ECS directions have been explored.

One of the most prominent strategies is to inhibit eCB degrading enzymes, MAGL and FAAH, in order to indirectly activate CB1 by 2-AG and AEA, respectively. In a study on rodents, URB602, a MAGL inhibitor (300–1000 pM injection), reduced NMDA infusion-induced panic effects via CB1- and CB2-dependent manner [111]. There is evidence on impact of FAAH activity on anxiety. FAAH overexpression in hippocampal CA1–CA3 glutamatergic neurons in a rodent model led to decreased AEA level in hippocampus, and resulted in anxiety-like behavior [112]. Certain FAAH genotypes result in increased activity of this enzyme, and in turn in reduced AEA levels [113,114]. Several recent studies target FAAH. URB597 (0.3 mg/kg), an FAAH inhibitor, proved to reduce anxiety in a CB1-dependent manner in a rodent model [115]. Another compound, PF-04457845 (4 mg/day), was tested in a human trial and showed an increase in recall of fear extinction memory and reduced negative effects of stress. Such molecules may be potentially used in the treatment of post-traumatic stress disorder (PTSD) [116]. Counterintuitively, FAAH overexpression in basolateral complex of amygdala (BLA) reduced anxiety-like behaviors. This may be connected with a lower AEA level increasing GABA transmission in BLA [117]. This is important information, as it shows the complexity of ECS and the importance of not only targeting a proper protein, but also its specific anatomical population.

ECS is also related to stress activation of hypothalamic-pituitary-adrenal axis (HPA). The administration of AEA (50 ng/5 μL intracerebroventricularly) proved to decrease levels of corticosterone in rodent model. In hypothalamus, both CB1 and CB2 were involved. In adrenal gland, AEA’s effects were caused by activation of TRPV1 [118]. Though CB1 antagonism usually leads to anxiety-like symptoms, it has been shown that CB1 antagonism in a specific region may have the opposite effect. Site specific CB1 antagonism (rimonabant—0.15 μg/0.1 μL) in the lateral habenula (LHb) reduced anxiety-like behavior in a rodent model [119].

### 4.4. Depression

ECS takes part in emotional regulation and, similarly to anxiety, downregulation of endocannabinoid signaling correlates with depressive behaviors [120]. The most important strategy to target ECS in depressive disorders is direct or indirect CB1 activation. WIN55,212-2, a CB1 and CB2 agonist, prevented anhedonia in a rodent model (0.5 mg/kg) [121]. New studies on ECS and depression show possible importance of CB2. Its deletion in dopaminergic neurons resulted in depressive behavior [122]. This opens up a possibility of targeting CB2 with agonists or using non-specific CB1 and CB2 agonists. Interestingly, a study on mice showed that the administration of either CB1 agonist ACPA (1 mg/kg), CB1 antagonist AM251 (1 mg/kg), CB2 agonist GP1a (2 mg/kg), or CB2 antagonist AM630 (0.5 mg/kg) had anti-depressive effects. Additionally, the co-administration of a small, ineffective dose of ketamine (5 mg/kg) with CB1 or CB2 antagonist led to antidepressant action [123]. Antidepressant effects of AM251 (10 mg/kg) had been shown before in rodent models [124]. It was suggested that antidepressant or depressogenic effects of CB1 antagonists may be dependent on time of administration of a drug, with chronic exposure favoring depression [120,125]. In addition, eCB degrading enzymes are targeted in depressive disorders. URB597 (0.3 mg/kg)—a FAAH inhibitor, prevented anhedonia in a rodent model [121]. Another FAAH inhibitor, PF3845 (10 mg/kg), decreased passive behavioral coping to acute stress in mice [126].

### 4.5. Addictions

ECS takes part in regulation of motivation and reward effects [77]. It is widely known that *Cannabis sativa* has a mild potential for addiction, caused mainly by activation of CB1 [127,128]. It may be both an inconvenience in drug design and a potential strategy for developing anti-addiction drugs.

CB1 inverse agonists may cause serious psychiatric adverse effects [11]. One of the possible ways to omit this problem is the design of CB1 neutral antagonists. AM4113 was tested in a rodent model of opioid addiction. It inhibited opioid self-administration, without rimonabant-like adverse effects (3–10 mg/kg/day) [129]. The same compound decreased also alcohol consumption in mice (1–3 mg/kg/day) [130]. Another prominent strategy is the development of CB1 allosteric modulators. ORG27569 (1.0–5.6 mg/kg), a CB1 NAM, reduced cocaine and methamphetamine seeking behavior in rats [131]. However, the exact mechanism of this action is unknown. Moreover, it has been shown that this compound may act in CB1-independent manner [62,132]. Thus, the utilization of CB1 NAMs in the treatment of addictions requires more research. A study on mice showed interactions between CB1 and ghrelin receptor, as administration of CB1 peripheral inverse agonist JD5037 (3 mg/kg/day) led to the reduction in alcohol intake. This was prevented in rodents lacking ghrelin or its receptor. It suggests the important impact of peripheral CB1 in healthy mammals on addictions. Therefore, CB1 peripheral antagonism emerges as a valid strategy with the reduced risk of central adverse effects [133].

On the other hand, CB1 agonists may also be helpful in some cases, for example in withdrawal syndromes [134], as chronic exposure to drugs of abuse impairs eCB transmission [77]. CB1 positive allosteric modulators may prove to be useful in this area. ZCZ011 (10–40 mg/kg), a CB1 PAM, attenuated Δ9-THC withdrawal somatic symptoms in mice [135]. Additionally, ECS activation within insula by JZL184 (10 mg/kg), a MAGL inhibitor, via CB1-dependent manner helped to relieve abstinence-related affective symptoms in a rodent model [136]. Interestingly, physical exercise has been shown to increase eCB levels, and thus may also be implemented in the treatment of withdrawal syndromes [137,138].

The second most prominent target in ECS for fighting addiction is the CB2. It was found, among others, in brain regions responsible for reward [139,140]. In the rodent model, the administration of CB2 antagonist, AM630 (1 mg/kg), increased ethanol intake, while CB2 agonist, JWH133 (1 mg/kg), had opposite effects [141]. Combining CB1 antagonism with CB2 agonism may be a reasonable strategy because of recent findings that opposing action on these two receptors has a synergistic effect on reward processing [142]. It has to be stressed that evidence for this approach is limited. However, it may be potentially beneficial to conduct more research on the co-administration of compounds with opposing actions on CBRs, and to consider combination drugs. Nevertheless, targeting CB2 requires caution. There is evidence that CB2 impact on addictions may be more complex. In a study on rodents, it was found that both CB2 agonism and antagonism reduced rewarding effects of alcohol, although through different mechanisms [143]. Therefore, more research is needed before considering CB2 as a safe target in the treatment of addictions.

### 4.6. Cognitive Functions

ECS involvement in cognitive functions is complex, and it is safe to say that ECS “regulates” learning and memory. There has been multiple evidence that CB1 activation impairs memory [144], while CB1 antagonists may enhance it [145]. For example, in traumatic brain injury (TBI), symptoms include impaired spacial learning and memory. This is connected with increased 2-AG levels. The administration of CB1 antagonist AM281 (3mg/kg) ameliorated aforementioned symptoms in mice [146]. The same compound (2.5–5 mg/kg) also improved cognitive deficits caused by scopolamine in the rodent model [147]. Rimonabant (1 mg/kg) improved memory in Down syndrome mice [148]. Pregnenolone (2–6 mg/kg), an endogenous CB1 NAM, reduced memory impairment caused by Δ9-THC administration in rodent models [149].

On the other hand, several studies prove that ECS signaling activation may enhance cognitive functions. Recently, a study showed that low doses of Δ9-THC (3 mg/kg/day) improved cognitive performance of old mice by enhancing gene expression, in which CB1 was crucial. It shows that activation of this receptor is an example of hormesis, with high doses of agonists exhibiting deleterious and low doses—a beneficial effect on cognitive functions. Moreover, in the case of the modulation of CB1 transmission, the age is also a very important factor [150]. Additionally, patients using medicinal *Cannabis* improved their cognitive performance after chronic administration of this plant. In addition, functional magnetic resonance imaging showed positive changes in their brain activation patterns. However, clinical relevance of these findings remains to be clarified, as there are several concerns, e.g., concentrations of Δ9-THC and CBD, and their influence on the outcome in comparison to the recreationally used *Cannabis* [151]. The odd positive impact of ECS activation on cognitive enhancement may be also due to CB2 anti-inflammatory action [152] or the impact of emotional state on learning and memory, all of which are modulated by ECS [153,154,155]. Other directions of enhancing memory or learning in the ECS mediated manner include MAGL or FAAH inhibition. Interestingly, MAGL inhibitor, JZL184 (0.5 mg/kg), enhances memory consolidation via CB2-dependent manner in rats [156]. The FAAH inhibitor, URB597 (0.3 mg/kg/day or 0.5 mg/kg intraperitoneally), does the same through both CB1 and CB2 activation in rodent models [154,157,158]. ECS’s possible influence on the nervous system is presented in Table 1.

## 5. Neurodegeneration

ECS takes part in the regulation of neurogenesis and neurodegeneration [161]. Most studies suggest that endocannabinoid signaling is usually beneficial in this context. CB1, CB2, and GPR55 take part in the regulation of neurogenesis. CB1 and GPR55 are crucial for proliferation and differentiation of neural stem cells (NSCs) [162,163,164]. Additionally, CB1 and CB2 control the development of astrocytes [165]. Therefore, CB1, CB2, and GPR55 agonists, as well as MAGL inhibitors may be worth being investigated in the context of neurogenesis, although, to date, there is no solid evidence to legitimize such path.

Endocannabinoid signaling may also be beneficial because of its impact on attenuating neurotoxicity and neuroinflammation. It has been shown that deletion of CB1 accelerates brain aging through tumor necrosis factor α (TNF-α) [166], while CB1 agonism decreases neuroinflammation [167]. There have also been studies on FAAH inhibition impact on decreasing neuroinflammation [168] and exitoxicity in rodent models [169]. Moreover, a GPR55 inverse agonist, KIT 17, in vitro inhibited PGE2 release in microglia (cells important for CNS immune functions), and thus may be further studied as a potential anti-neuroinflammatory agent [170]. As a system important for neuronal growth and death, ECS has been targeted in studies regarding Alzheimer’s, Parkinson’s, and Huntington’s diseases, and others, such as amyotrophic lateral sclerosis (ALS).

In Alzheimer’s disease (AD), it has been shown that CB1 deletion worsens the symptoms [171]. CB1 agonist, ACEA (3 mg/kg), exhibited anti-apoptotic action on neurons and reduced cognitive impairment in the AD rodent model [172]. Lipoxin A_4_, an endogenous CB1 positive allosteric modulator, was shown to have protective properties against β-amyloid-induced spacial memory impairment in mice [173]. Due to its presence in microglia, CB2 may also be a promising target [174]. Interestingly, CB2 forms heterodimers with GPR18, which exhibit considerable interactions, thus GPR18 may be a promising target, although more research is needed [175]. Moreover, β-amyloid promotes MAGL overexpression. Therefore, increasing 2-AG concentration via MAGL inhibition has therapeutic potential in AD. Indeed, MAGL inhibitor forsythiaside in vitro decreased neuroinflammation via CB1-dependent nuclear factor-κB (NF-κB) pathway [176]. TRPV1 agonist, capsaicin, reduced β-amyloid-induced degradation of hippocampal neuron functions in vitro [177].

As ECS modulates nigrostriatal pathway and dopaminergic transmission, it may find its place in Parkinson disease therapy. Targeting CB1 and GPR55 was proposed [178]. In addition, FAAH inhibitor, URB597 (0.2 mg/kg every 3 days for 30 days), was shown to decrease dopaminergic neuronal death and improve motor functions in the rodent exitoxicity model [179].

In addition, CB1 is downregulated in Huntington’s [180,181]. GAT229 (10 mg/kg/day), a CB1 PAM, positively affected motor functions and gene expression, as well as delayed the onset of symptoms in the mice model of Huntington’s disease [182]. MAGL inhibitor, JZL-184 (18 mg/kg, intraperitoneally), was shown to restore dopaminergic signaling in CB1-dependent way, and thus normalize behavior in mouse model [183]. ECS targeting is also proposed in treatment of spinocerebellar ataxias (SCAs), although there is some contrary evidence. CB1 agonism is believed to prevent neurotoxicity [184]. On the other hand, some older studies indicate that enhancing CB1-dependent signaling may affect in ataxic symptoms [185,186]. CB2 activation is proposed for neuroprotection [187].

FAAH inhibitor, PF3845, attenuated development of human immunodeficiency virus 1 (HIV-1) associated neurocognitive disorders (HAND) in CB1- and CB2-dependent manner in vitro. CB1 activation was proved to decrease intracellular Ca levels, thus decreasing neurotoxic glutaminergic activity. CB2 activation was responsible for reduction of neuronal death and degradation [188].

While CB1 agonism is usually the proper direction in fighting neurodegenerative diseases, there are exceptions—for example, in focal cortical dysplasia (FCD), where upregulation of CB1 leads to increased mTORC1 (mammalian target of rapamycin complex 1) signaling, and thus to malformations in cortical development. Rimonabant was proved to decrease mTORC1 overactivation in vitro [189].

## 6. Inflammatory and Autoimmune Diseases

CB2 is distributed mainly in cells of the immune system [9]. Therefore, it is natural that this receptor has been viewed as a potential target for the treatment of inflammatory and autoimmune diseases. There are numerous studies proving that CB2 activation leads to anti-inflammatory action. In various mammalian models, CB2 agonists inhibit recruitment of leukocytes, and decrease levels of TNF-α and interleukin 1β (IL-1β) [190], IL-6, IL-18, monocyte chemoattractant protein 1 (MCP-1) [191], and reactive oxygen species (ROS) [192]. Additionally, CB2 agonism results in 5′ AMP-activated protein kinase (AMPK) activation and subsequent reduction in anabolic processes and promotion of oxidative phosphorylation (OXPKOS), and thus anti-inflammatory effect [193,194,195]. CB2 agonists may be employed in the treatment of rheumatoid arthritis, atherosclerosis, inflammatory bowel disease [192], and ocular inflammations [196].

On the other hand, CB2 antagonists may be of use as well. Lipopolysaccharides (LPS, endotoxins) are molecules on the membrane of Gram-negative bacteria. Normally, LPS exposure leads to inflammatory response, among others, in the form of increased TNF secretion. Prolonged exposure to LPS results in toll-like receptor 4 (TLR4) activation, subsequent increase in 2-AG production in mast cells, CB2 activation, and finally hyporesponsivity of mast cells, called endotoxin tolerance (ET). CB2 antagonist, AM630, prevented ET in vitro [197], and thus CB2 antagonists may be studied further as a potential treatment for immunoparalysis.

Apart from CB2, also other components of ECS take part in the regulation of immune processes. Their impact is complex, in part because of interdependence of ECS and the prostanoid system [198]. AEA and PEA usually exhibit anti-inflammatory effects, while 2-AG may act both anti- or pro-inflammatory [199,200]. Alongside CB2, CB1 also contributes to the homeostasis of the immune system. Unfortunately, its functions are not straightforward. Increased expression of CB1 was shown to promote oxidative stress and inflammation [201]. Contrarily, CB1 agonists decreased mast cell activation in vitro, exhibiting also anti-inflammatory activity in the rodent model, and are proposed as anti-inflammatory agents in psoriasis and dermatitis [202]. Moreover, as we showed above, CB1 agonists exhibit anti-neuroinflammatory properties. In addition, the MAGL inhibitor JZL184 (4mg/kg) reduced TNF-α levels in a CB1-dependent manner in mice [203]. FAAH inhibitors proved to be anti-inflammatory agents in vitro and in rodent models. In detail, they reduce PGE2 production, downregulate COX-2, reduce expression of pro-inflammatory cytokines, reduce inducible nitric oxide synthase (iNOS) activity, and alleviate TLR3 mediated fever [168,204,205]. The role of TRPV1 remains unclear. Some studies suggest that TRPV1 activation leads to the release of pro-inflammatory cytokines in asthma, and TRPV1 antagonists could be used in the treatment of this disease [206]. Other publications show that TRPV1 agonists reduce TNF-α production in rodent models and may be employed in inflammatory diseases [207]. Additionally, GPR55 seems to be a promising target. AEA was shown to inhibit mast cells degranulation in vitro via CB2 and GPR55 activation, and CB2-GPR55 heterodimers were probably involved [208]. On the other hand, GPR55 antagonists exhibited anti-neuroinflammatory action in vitro [170].

ECS proteins may also be used in the treatment of autoimmune diseases. Dual CB2/PPARγ agonists, JBT-101 (5 mg/kg), and EHP-101 (a lipidic formulation of VCE-004.8) (20 mg/kg) alleviated skin fibrosis in rodent models of systemic sclerosis (SSc). Moreover, JBT-101 (ajulemic acid, Lenabasum) (5–20 mg) was shown to exert anti-inflammatory action in a human skin inflammation study [209,210]. JBT-101 reached phase III or II clinical trials for several indications (see: Section 11. Approved drugs and clinical trials). In addition, CB2/PPARγ agonist with CB1 antagonistic property, VCE-004.3, was proposed for the treatment of SSc, based on promising in vitro results [211]. Dual CB1/iNOS inhibitor, MRI-1867 (10 mg/kg), exhibited antifibrotic activity in the rodent model of pulmonary fibrosis [212]. Alteration in ECS proteins expression was shown in psoriasis, with an increase in CB1 in psoriatic arthritis, CB2 in psoriasis vulgaris, and GPR55 in both forms of the disease. In addition, elevated levels of 2-AG and AEA were observed [201]. Therefore, ECS seems to be a reasonable target in psoriasis, although more research is needed. Surprisingly, CB2 activation was found to play a pathogenic role in renal fibrosis. XL-001 (20 mg/kg intraperitoneally), a CB2 inverse agonist designed using in silico methods, reduced inflammation and ameliorated kidney fibrosis in the rodent model [213].

## 7. Metabolic Diseases

One of the main functions of ECS is the control of energy homeostasis. Endocannabinoid signaling favors energy intake and storage, as well as affects metabolism and thus it can contribute to the development of obesity and metabolic syndrome [214].

### 7.1. Obesity

Cannabinoids are known to increase appetite, even in a satiated state, and the consumption of highly palatable food [77]. This orexigenic effect is mediated via CB1 activation in forebrain glutamatergic neurons, hypothalamus, and in a mesolimbic dopamine system [215]. Thus, a pharmacological modulation of CB1 may be used to control food intake [216].

In spite of CNS-supervised mechanisms, ECS also participates in the peripheral control of energy metabolism. First of all, CBRs regulate the conversion of white adipose tissue (WAT) into thermogenic, positively influencing energy balance beige adipose tissue in process called browning. This transformation is enhanced either by CB1 inhibition or CB2 activation [214]. Moreover, the CB1 activation promotes adipogenesis, liver lipogenesis, and may exert a pro-inflammatory effect [215] while CB2 mediates inhibition of obese-related inflammation and thus decreases the risk of adverse outcomes in obesity [214].

Considering this, CB1 antagonists or inverse agonists as well as CB2 agonists seem to be a useful tool in the treatment of obesity and related symptoms. Indeed, rimonabant (20 mg/day) proved to be efficient in reducing body weight and in improving cardiovascular and metabolic risk factors in phase III clinical trial [217]. However, its severe psychiatric adverse effects suggest that the central CB1 inverse agonism is not the right direction. Peripheral antagonism of CB1 and thus avoiding central effects appears to be a reasonable option. Recently, significant research has been conducted on peripherally restricted CB1 antagonists or inverse agonists [218]. For instance, AJ5012 (10–20 mg/kg/day), a peripheral CB1 antagonist, was reported to cause a significant weight loss, increase energy expenditure, ameliorate glycemic control and insulin sensitivity, and to reduce inflammation in rodent model [219]. Similarly, AJ5018 (10 mg/kg/day) was shown to improve metabolic abnormalities and suppress adipose tissue inflammation via moderation of macrophage infiltration, activation of the NLRP3 inflammasome, and reducing production of pro-inflammatory cytokines in mice [220]. Neither compound elicits neurobehavioral adverse effects due to low brain penetrance. Other possible strategy is the employment of CB1 neutral antagonists instead of inverse agonists. Such compounds (e.g., PIMSR—10 mg/kg/day) were shown to effectively reduce body weight and adiposity in mice and may be safer in terms of psychiatric adverse effects [221]. Moreover, CB1 NAMs have been proposed as well. PSNCBAM-1 (30 mg/kg intraperitoneally) significantly reduced food intake and decreased body weight in a rodent model [222]. Administration of ORG27569 (5.6–10 mg/kg/day) also led to hypophagic effect in mice [223]. However, more research is needed, as it was shown that ORG27569 in some cases may act in a CB1-independent manner [62,132].

In addition, GPR55 participates in the regulation of energy metabolism. Its deficiency was found to significantly decrease insulin signaling in adipose tissue, liver, and skeletal muscle, reduce physical activity, and enhance adiposity via increased lipogenic proteins expression in adipose tissue in rodents [224]. Recently, dual GPR55 and GPR18 agonists, such as O-1602 (5 mg/kg/day) and O-1918 (1 mg/kg/day), were shown to improve albuminuria and decrease body weight and body fat in rats, unfortunately, also causing changes in liver and kidney morphology and increased inflammation [225].

### 7.2. Diabetes

Apart from the ECS role in obesity, ECS deregulation contributes to occurrence and progression of type 2 diabetes mellitus (T2DM) [226,227]. Considering CB1 participation, its activation was shown to decrease insulin signaling, reduce proliferation and viability of pancreatic β-cells, and to promote pancreatic inflammation [228,229]. CB1 antagonists (e.g., JD5037—3 mg/kg) were shown to reverse diabetic neuropathy (DN) in mice via modulation of glucose transporter 2 (GLUT-2) expression and activity in renal proximal tubule cells [230,231]. Furthermore, AM6545 (10 mg/kg/day), a peripheral CB1 antagonist, combined with angiotensin-converting enzyme (ACE) inhibitor, was shown to reverse albuminuria, nephrin loss, and to inhibit inflammation in rodent model of DN [232]. In addition, suppressing CB1 expression by microRNA-29a decreased pro-inflammatory and pro-fibrogenic mediators’ levels as well as mitigated renal hypertrophy in mice [233].

On the contrary, CB2 generally exerts the opposite effect on diabetes and DN. The CB2 activation enhances insulin release, decreases pro-inflammatory cytokines’ levels, and attenuates oxidative stress [234]. In addition, activation of CB2 by HU308 (3 mg/kg/day) exerted a cardioprotective effect in diabetic cardiomyopathy in mice [235].

Other GPCR, GPR55 elicits a beneficial impact on T2DM due to increasing insulin secretion, improving glucose tolerance, and a positive effect on β-cells’ viability [229]. For instance, GPR55 agonists, such as O-1602, were demonstrated to reduce endoplasmic reticulum (ER) stress-induced apoptosis in pancreatic β-cells in vitro [236].

It is interesting that GPR119 enhances both insulin and glucagon-like peptide-1 secretion [237,238]. A significant number of GPR119 agonists took part in clinical trials, from which the majority was discontinued [239]. However, multi-target drugs may be a promising strategy, as a dual GPR119 agonist and dipeptidyl peptidase 4 (DPP4) inhibitor, HBK001 (30 mg/kg), was demonstrated to meaningfully improve glucose homeostasis and β-cell function in the rodent model [240].

### 7.3. Hepatic Diseases

ECS partially controls hepatic glucose and lipid metabolism as well as influences fibrogenesis and inflammation. Thus, ECS members, especially CB1 and GPR119, are promising targets in the treatment of liver diseases [241]. While CB1 promotes fibrogenesis, activation of CB2 results in antifibrogenic responses [241]. CB1 inhibition with rimonabant (10 mg/kg/day) in the mice model of nonalcoholic fatty liver disease (NAFLD) improved adipokine profile, decreased glucose plasma concentration, and reduced inflammation in adipose tissues and liver [242] and in the rat model of nonalcoholic steatohepatitis (NASH) inhibited hepatic fat infiltration, inflammation, fibrogenesis, and cellular death [243]. In addition, APD668 (30 mg/kg), a GPR119 agonist, was shown to reduce circulating cholesterol, glucose, triglyceride, and hepatic injury markers in rodent NASH model [244]. Therefore, GPR119 agonists may be useful for the treatment of dyslipidemia and NASH.

## 8. Cardiovascular Diseases

According to the latest systematic review, *Cannabis* use may result in cardiovascular adverse effects, including ischemic stroke [245]. Nonetheless, plenty of studies reported that the modulation of ECS results in the alleviation of hypertension, atherosclerosis, myocardial ischemia, and related diseases [246].

### 8.1. Hypertension

The ECS control of blood pressure (BP) involves various neuronal and non-neuronal mechanisms. Based on various human and animal studies, CB1 is suggested to play a major role in these actions and while central activation of CB1 increases BP, peripheral activation tends to lower BP [246,247]. However, the activation of either or both CBRs was demonstrated to increase contractility in vascular smooth muscles in vitro [248].

Cannabinoids, acting peripherally, are generally known to decrease BP. However, the final outcome depends on the type of a cannabinoid ligand, model of hypertension as well as age and sex [247]. For instance, AEA (5 mg/kg/week) decreased BP and reversed all altered cardiovascular markers and parameters in hypertensive rats [249].

Lately, FAAH inhibitors are investigated as antihypertensive drugs. PF-3845 (0.45–0.9 μM/kg), a selective FAAH inhibitor, reduced mean arterial pressure and increased diuresis in mice [250]. Another FAAH inhibitor, URB597 (1 mg/kg twice a day), lowered BP and heart rate in older but not in younger rats [251]. However, special caution must be taken in drugs targeting FAAH, as URB597 (1 mg/kg twice a day) was reported to disturb the kidney redox system as well as phospholipid ROS-dependent and enzymatic-dependent metabolism in the rodent model [252].

In addition, using TRPV1 agonists may be an interesting way to modulate BP centrally as TRPV1 in ventral medial prefrontal cortex was reported to facilitate the cardiac baroreflex response in rats through stimulating the NMDA activation and NO synthesis. [253]. However, based on in vitro study, TRPV1 activation may aggravate idiopathic pulmonary arterial hypertension (IPAH) [254].

### 8.2. Atherosclerosis

As ECS is known to regulate inflammatory and metabolic processes, its modulation seems to be useful in atherosclerosis. ECS components are suggested to modulate vascular inflammation, leukocyte migration, macrophage cholesterol metabolism, and plaque stability [255].

CB1 activation generally exerts a deleterious effect in atherosclerosis. CB1 agonists such as AEA or HU-210 promoted endothelial dysfunction via ROS generation and promotion of apoptosis in vitro [256]. Moreover, rimonabant (8 mg/kg/day) decreased atherosclerotic lesions in the aorta and increased serum adiponectin levels in mice [257]. On the contrary, CB2 activation exerts atheroprotective actions due to its anti-inflammatory and anti-proliferative properties [255], and this may be achieved via MAGL inhibition. MAGL genetic deficiency or inhibition with JZL184 (16 mg/kg 3 times a week) were shown to limit plaque formation in mice. Furthermore, the CB2 activation by 2-AG mediated atheroprotective B1a lymphocyte phenotype [258].

GPR55 agonism might be an interesting strategy to modulate arterial inflammation [255]. For instance, PEA (3 mg/kg/day), acting via GPR55 and PPARα in early atherosclerosis stabilized plaque in pre-established phase of the disease in mice. GPR55 activation promoted anti-inflammatory and pro-resolving macrophage subtype [259]. In addition, GPR55 role in regulating neutrophil degranulation in atherosclerosis was demonstrated in rodent model by the use of GPR55 antagonist CID16020046 (0.5 mg/kg 5 times a week) [260].

### 8.3. Myocardial Dysfunctions

The data on cardioprotective impact of endocannabinoids are conflicting and are based mainly on studies on rodents. Some studies suggest 2-AG deleterious impact in myocardial infraction (MI) via CB2 activation and myeloid cells recruitment, as shown by the MAGL inhibitor, JZL184 (16 mg/kg) in mice [261]. According to another study on rodents, the CB2 activation promotes myocardial adaptation to pressure overload and remodeling via apoptosis prevention, inflammation and oxidative stress reduction, and regulation of contractile elements’ expression in cardiomyocytes [262]. Furthermore, according to several in vivo studies, activation of CB2 and TRPV1 modulates myocardial damage during myocardial ischemia [246].

MAGL inhibition gives promising results in the case of cardiac arrest. Administration of single dose of URB602 (5mg/kg), a MAGL inhibitor, turned out to significantly improve survival rate and reduce myocardial injury in the rodent model [263].

## 9. Cancer

**Figure 3:**
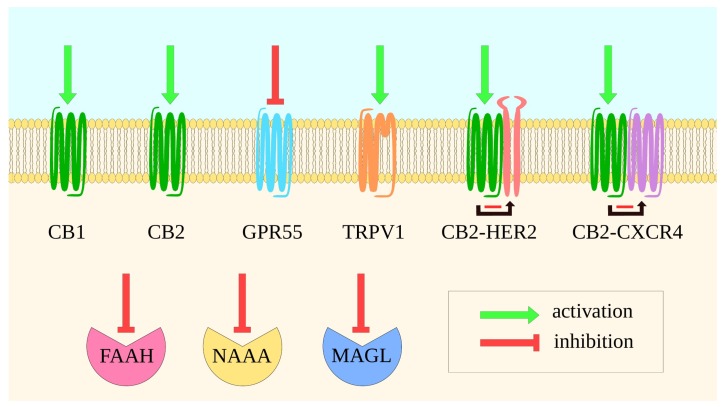
A schematic presentation of ECS components targeted in cancer. The figure shows typical and new directions of ECS modulation in neoplasm diseases. Well-investigated directions include the activation of CBRs or TRPV1, suppression of GPR55 signaling as well as the inhibition of FAAH and MAGL. Promising targets may be another cannabinoid degrading enzyme, NAAA [264] as well as CB2 heteromers, such as CB2-HER2 and CB2-CXCR4, as activation of CB2 inhibits HER2 and CXCR4 signaling, respectively [265,266]. Green arrows indicate protein activation, red arrows—inhibition. Black arrows with red lines show HER2 or CXCR4 inhibition by CB2 activation in respective heterodimers.

Although, to date, the use of cannabinoids in cancer is limited to alleviating symptoms like nausea or pain, a plethora of studies revealed both their antitumor and protumorigenic properties. As most components of ECS are overexpressed in tumor cells, its modulation appears to be a promising approach, as shown in Figure 3.

Multiple CB1 and CB2 agonists were shown to elicit antitumor properties both in vitro and in vivo (for a review, see: [267]). For instance, CB2 agonist, LV50 elicited cytotoxic effect in lymphoblastoid cells with high specificity to neoplasm cells [268], and WIN 55,212–2, CB1/2 agonist was able to arrest cell cycle and cause apoptosis in renal carcinoma in vitro [269]. In addition, allosteric CB1 modulators inhibiting cell proliferation in vitro [270] are a promising approach. Such compounds may allow for avoiding CB1 activation-related adverse effects. However, there is a report on tumor-progressive roles of CB1 agonist in melanoma cells [271].

CB2 may exist in heteromers in various cancer subtypes. CB2 forms heteromers with human epidermal growth factor receptor 2 (HER2) in HER2+ breast cancer cells. CB2 regulates HER2 signaling, leading to a prooncogenic effect. However, the CB2 activation with an exogenous agonist disrupts the HER2 protumoral action in vitro [266]. CB2 can be found in connection with C-X-C chemokine receptor type 4 (CXCR4) in breast and prostate cancer cells. In vitro, CB2 activation was shown to reduce CXCR4-mediated cancer functions such as chemotaxis [265].

Concerning eCBs, they generally exert tumor-suppressive effect, based on clinical observations [272]. In vitro, AEA was demonstrated to decrease invasion and metastasis by upregulating a tissue inhibitor of matrix metalloproteinases-1 (TIMP-1) [273] and to induce apoptosis via oxidative stress in COX-2- [274] and TRPV1-dependent manner [275]. 2-AG (20 mg/kg/day) showed similar properties in the rodent model, at the same time suppressing tumor immune-mediated surveillance [276]. Therefore, inhibition of degrading enzymes and thus upregulation of eCBs’ levels seems to be a valid strategy.

High levels of MAGL were shown to be associated with tumor malignancy, based on clinical observations and in vivo studies [277,278,279]. Concerning pathomechanism of MAGL, formation of protumorigenic lipid signals rather than lowering eCBs’ levels causes procancerogenic effects [280]. Recent research is focused on highly selective reversible MAGL inhibitors with anticancer properties [281]. For instance, JZL184 was reported to reduce cancer cells growth, induce apoptosis, and prevent invasion in vitro [279,282] as well as suppress osteolytic bone metastasis and alleviate cachexia and bone mass loss in rodents (8–16 mg/kg) [283]. Another MAGL inhibitor, URB602 (5mg/kg), suppressed progression of colon cancer in mice [284].

FAAH inhibitors were also proved to exert antiproliferative and proapoptotic action in multiple in vitro studies, especially in combination with ethanolamines [285]. Furthermore, there appeared a proposition of development of dual FAAH inhibitors and PPAR agonists as a multi-target approach in anticancer therapy [286]. In addition, 1,2-dihydro-2-oxo-pyridine-3-carboxamides, influencing at least two components of ECS, showed cytotoxic activity in lymphoblastoid cells [287].

N-acylethanolamine acid amidase (NAAA), another eCB degrading enzyme, was reported as a promising tool in cancer therapy, as NAAA inhibitors reduced bladder cancer cells proliferation and inhibited migration in vitro [264].

TRPV1 is extensively investigated in neoplasms. Its stimulation with AEA or CBD led to apoptosis and inhibited proliferation of different types of tumor cells [275,285].

GPR55 appears to be a promising target as well. Its activation with lysophosphatidylinositol (LPI) was demonstrated to elicit a tumor promoting effect in colon (in vitro) [288], colorectal (in vitro and in vivo) [289], and breast carcinoma (in vitro and in vivo) [290,291]. In this spotlight, LPI-GPR55 axis inhibitors may be an effective strategy to suppress cancer progression. Moreover, a GPR55 peptide ligand was shown to suppress the proliferation of B-lymphoblastoid cell lines [292].

In addition, combining cannabinoids with other anticancer therapies brings enhanced results [293]. For example, in the case of triple negative breast cancer, the combination of photodynamic therapy with CB2 agonist (JWH-133) (40 nmol) resulted in synergistic inhibition of tumor growth and extended survival time in mice [294].

## 10. Other

### 10.1. Respiratory Disorders

The data on the role of ECS in asthma are ambiguous. On the one hand, the *Cannabis* use is known to elicit deleterious effects on the respiratory tract and to exacerbate asthma symptoms [295]. On the other hand, targeting CBRs may be effective in asthma treatment due to bronchodilatory action. This effect is caused by CB1 activation and subsequent inhibition of release of acetylcholine and other mediators from parasympathetic terminals in bronchi [296]. In addition, the CB1 activation by ACEA (7.5 mg/kg/day) prevents 5-HT-induced tracheal hyperactivity in mice [297]. Furthermore, CBD (5–10 mg/kg) was recently reported to decrease the inflammatory and remodelling processes in a murine model of allergic asthma probably via CB1/CB2 signaling and in receptor-independent manner [298].

CBRs are also involved in pulmonary fibrosis. Studies showed that CB1 exerts proinflammatory and profibrotic effect in pulmonary fibrosis [299,300] and peripherally restricted dual inhibitor of CB1 and iNOS, MRI-1867 (10 mg/kg/day), partially attenuated fibrosis in rodent models [301]. Concerning CB2, its agonist, JWH 133 (1 mg/kg), attenuated nicotine-induced fibrosis while AM630 (0.5 mg/kg), a CB2 antagonist, enhanced it in mice [302].

### 10.2. Gastroenterology

ECS controls gastrointestinal (GI) tract (GIT) motility, sensitivity, and inflammatory processes, and thus appears to be an interesting target in the treatment of GI diseases, with a significant role in inflammatory bowel disease (IBD) [303]. Although the data on ECS tone in IBD is not consistent, the eCBs level is generally increased, from which LPI, an endogenous GPR55 agonist, may exert a pro-inflammatory effect, based on clinical observations [304]. A number of CB1 and CB2 agonists were proven to be effective in ameliorating IBD symptoms in animal models. However, clinical studies on THC and CBD in IBD treatment in most cases failed to meet the primary endpoint, but an improvement was observed [305]. According to recent research, AM9405 (0.1–1 mg/kg), a peripheral CB1 and 5-HT3 agonist, suppressed hypermotility of the GI tract and reduced pain in the rodent model [306]. In terms of other GI disorders, a retrospective human study showed that nabilone (1–10 mg/kg), a CB1 agonist, efficiently reduced diarrhea and induced weight gain showing a reasonable safety profile [307]. In another study, a CB1 positive allosteric modulator, ZCZ011 (10–40 mg/kg), combined with an ineffective dose of MAGL inhibitor JZL184 (1 mg/kg intraperitoneally), exhibited antiulcerogenic properties in a rodent model [135]. In addition, inhibition of eCBs degrading enzymes is a promising approach, as numerous FAAH inhibitors and one MAGL inhibitor were shown to reduce IBD symptoms in animal models [305].

Endocannabinoid signaling is also known for its anti-emetic properties. This is associated mostly with an activation of both central CB1 and peripheral CB1 in GIT. Thus, CB1 agonists may act as anti-emetic agents. On the other hand, CB1 inverse agonists exert emetogenic properties, as shown by the example of rimonabant in clinical trials [308]. Cannabinoids are also potent solutions for chemotherapy-induced nausea that is unresponsive to traditional drugs (see: Section 11. Approved drugs and clinical trials) [309]. Moreover, CB2 and TRPV1 agonists are also proposed as anti-emetic drugs, based on results of animal studies. [308,310].

### 10.3. Osteology

ECS is involved in osteoclast/osteoblast activity with the emphasis on CBRs and TRPV1 function. CB1 activation generally inhibits osteogenic signaling [44]. In addition, the downregulation of CB1 expression in hypothalamus by central adiponectin in the epigenetic mechanism was reported to promote peripheral bone formation in mice [311]. Importantly, the action of CB1 antagonists is age-dependent: rimonabant (0.2 mg/kg) prevented osteoporosis in young rats but aggravated it in older ones [312].

On the contrary, CB2 activation promotes bone matrix deposition. For instance, CB2 agonists, HU-308, and its more potent enantiomer HU-433 were shown to stimulate osteoblast proliferation and osteoclast differentiation in vitro [313].

TRPV1 activation leads to osteoclasts differentiation in the RANKL-mediated pathway. TRPV1 antagonist, SC0030, inhibited this process in vitro [314]. Considering this, dual CB2 agonists and TRPV1 antagonists are proposed as a promising tool in bone mass loss treatment [44].

### 10.4. Reproductive System

Concerning female reproductive system, ECS affects the hypothalamic-pituitary-ovarian (HPO) axis, steroid hormones production, and secretion, as well as controlling ovaries and uterus functioning. Changes in ECS in female reproductive tissues may cause pathological states [315]. For example, based on clinical observations, increased AEA plasma level and decreased FAAH endometrium level are probably involved in pathogenesis of polycystic ovarian syndrome [316]. CBRs may also be associated with adenomyosis, as their expression is significantly lowered independent on cycle phase in endometrium of adenomyosis bearing patients [317]. CB1 agonists are investigated as a possible treatment for endometriosis with contradictory results. While WIN 55,212-2 limited cell proliferation in vitro, methanandamide (5 mg/kg) contributed to development of ectopic lesions in mice [318]. However, modulation of ECS in gynecological disorders has lot of limitations. ECS is activated with spatial and temporal specificity, plasma, and local levels of ECS components often differ and mechanisms underlying ECS regulation of female reproductive system and the crosstalk with HPO axis are not fully revealed [315].

When it comes to male reproductive system, CB1 is present in nerves in corpus cavernosum [319] and is responsible for *Cannabis*-related erectile dysfunction [320]. While designing CB1 agonists, one may expect possible sexual adverse effects. On the other hand, CB1 antagonists were proposed for the treatment of erectile dysfunction, e.g., SR 141716A (1 mg/kg twice a day), interestingly exhibiting action via central CB1 in hypothalamus, based on a study on rodents [321]. Due to the localization of CB1 population important in this context, the CB1 peripheral antagonists or inverse agonists may be a valid strategy as well.

### 10.5. Dermatology

Members of ECS regulate key aspects of skin homeostasis, including immune modulation, inflammation, cell proliferation, and differentiation as well as regulation of sebaceous glands, hair growth, pigmentation, and wound healing and thus its dysregulation contributes to various cutaneous diseases (for a review, see: [322,323]). According to a recent study, inhibition of eCB reuptake in human sebocytes with VDM11 exerts an anti-inflammatory effect as well as elevates sebaceous lipid production and eCB levels. This may be beneficial in treatment of skin conditions accompanied with inflammation and dryness [324].

### 10.6. Genetic Disorders

Targeting CB1 may be an interesting approach to treat genetic disorders like Duchenne muscular dystrophy. CB1 expression is upregulated on the onset of this disease. As CB1 signaling inhibition by rimonabant resulted in the promotion of human satellite cell differentiation in vitro, increase of muscle regeneration, and improvement of locomotor activity in dystrophic mice (0.5 mg/kg 3 times a week), CB1 antagonism appears to be useful in treatment of muscular dystrophies [325]. It is interesting that CBD and cannabidivarin (CBDV) elicit similar effects [326].

## 11. Approved Drugs and Clinical Trials

We described a plethora of possible therapeutic areas, in which ECS proteins may potentially be targeted. The advancement of the aforementioned research varies. Many of these proposals are supported by limited evidence. While it may be worth exploring some of them more deeply, and potentially to employ such ideas in the future drug design, many of these directions will probably turn out to be a blind alley. However, there are also drugs and drug candidates with solid information about their action and safety verified by clinical trials (Table 2).

Several drugs reached the market. These include mainly THC, CBD, and their analogs or combinations. Drugs containing dronabinol (Δ9-THC) were approved by the Food and Drug Administration (FDA) for 1) nausea and vomiting associated with cancer chemotherapy in patients who have failed to respond adequately to conventional antiemetic treatments, and for 2) anorexia associated with weight loss in patients with acquired immunodeficiency syndrome (AIDS) [309,327,328,329]. Cesamet, a drug containing THC analog–nabilone, also was approved for the first aforementioned indication [330,331]. Alas, administration of THC and its derivatives alone comes with risks of adverse effects, as such drugs have a narrow therapeutic window [332]. However, combination of THC, a CB1/2 partial agonist, with CBD was shown to attenuate THC-derived psychiatric adverse effects [333], which is also important in the case of the proportions of these compounds in *Cannabis* [334]. Combinations of THC and CBD are approved in multiple countries for the treatment of spasticity and pain in multiple sclerosis or cancer pain [335,336]. In addition, CBD alone has its place in medicine. Epidiolex, an oral solution containing CBD, was approved by FDA in two forms of epilepsy: Lennox–Gastaut syndrome and Dravet syndrome [100]. CBD has a complex mechanism of action, including CB1 negative allosteric modulation, TRPV1 agonism, GPR55 antagonism, and 5-HT1A agonism, and interacts with multiple other pharmacologically relevant receptors and channels [40,101,102,103]. Other examples of drugs that reached the market include Acomplia or Zimulti, containing rimonabant, a CB1 inverse agonist, as an active ingredient. They were approved in Europe for the treatment of obesity but were withdrawn due to major phychatric adverse effects [10]. These examples show that drugs or combinations of drugs targeting various ECS proteins may exhibit more desirable therapeutic profiles than compounds acting on a single molecular target. Such strategy might be valid in the context of not disrupting the homeostasis of ECS. However, the combination of THC and CBD is the most extensively studied example of this direction. In order to work on other potential ECS-related multi-target drugs, the ECS itself has to be better understood, which includes exploring multiple effects of the endocannabinoid signaling modulation. Moreover, CB1 activation or inhibition has to be planned with caution, as aside from desired effects, it may also lead to adverse ones, especially due to the impact on central CB1.

Because of a great potential of targeting ECS, new potential drugs are constantly developed. Some of them are being tested in clinical trials. Among them, CB2 agonists reached the most advanced phases. Because this receptor is distributed mainly in the immune cells [9], CB2 agonists were naturally investigated as a potential treatment for autoimmune and inflammatory conditions. JBT-101 (Lenabasum) is currently in phase III trials for diffuse cutaneous systemic sclerosis and for dermatomyositis, and in phase II trials for cystic fibrosis, systemic sclerosis, and systemic lupus erythematosus [337,338]. APD371 (Olorinab) reached phase II trials for abdominal pain in Crohn’s disease and for irritable bowel syndrome [339].

As targeting CB1 comes with risks of central adverse effects, some prospective drugs were designed to overcome this issue by acting only on peripheral CB1. NEO1940 (ART27.13) is a peripheral CB1 and CB2 agonist with a phase II trial planned for cancer related anorexia. Phase I trials showed desired weight gain [340]. CRB-4001, a peripheral CB1 inverse agonist is waiting for phase I trial as a potential drug for nonalcoholic steatohepatitis [341,342].

Other proteins of ECS are targeted by compounds that reached clinical trials as well. In the area of eCB degrading enzymes, ABX-1431, a MAGL inhibitor is the most promising drug candidate. It reached a phase II trial for the treatment of Tourette’s syndrome and chronic motor tic disorder [343].

Several FAAH inhibitors were tested in clinical trials as prospects for new analgesic drugs. A few examples include PF-04457845 [344], JNJ-42165279 [345], and BIA 10-2474 [346]. Unfortunately, all of them reached at most phase II. They proved to be either not effective or led to neurological disorders. Causes of the undesirable therapeutic profile of FAAH inhibitors remain not entirely clear. The proposed explanations suggest the pro-nociceptive activity due to the activation of presynaptic TRPV1 by AEA, and AEA being metabolized to pro-inflammatory prostaglandins by COX. As the up-to-date failures show, FAAH inhibition seems to be a risky direction in drug design. Possible solutions to overcome the obstacles, and to utilize FAAH-targeting compounds in medicine include dual FAAH/TRPV1 and FAAH/COX-2 inhibitors, although these proposals require more evidence [84].

NEO6860, a TRPV1 antagonist, was tested for osteoarthritis pain in a phase II trial. Thus far, it did not exhibit desired effects in comparison to placebo [347]. However, phase I trials showed analgesic activity. Moreover, NEO6860 administration did not lead to hyperthermia, which is a major problem in the case of TRPV1 antagonists [159,348]. Thus, further studies on this compound may be potentially rewarding.

## 12. Discussion

ECS is indisputably a complex system. Targeting it has a lot of potential for drug design. Possible diseases and disorders with specific ECS targets that were widely described in this review are concluded in Supplementary Table S1. However, there are some important difficulties, which we will highlight with the example of CB1, and gather in one place the risks that come with targeting this protein with the possible solutions, both of which we discussed in various parts of this review. As CB1 is the main receptor of ECS, a lot of studies were directed to understanding its action, and today it is the ECS protein we have the most comprehensive knowledge of. Firstly, the central activation of CB1 comes with the risk of cognitive impairment [75] and disrupting the ECS regulation of the reward system [76,77]. To avoid these adverse effects, peripheral CB1 agonists, CB1 positive allosteric modulators or indirect activation of CB1 via MAGL or FAAH inhibition are usually proposed. Additionally, the CB1 activation may be associated with weight gain [77], inflammation [201], and erectile dysfunctions [320]. Secondly, the CB1 inverse agonism affects in depression, anxiety [10], and nausea [308]. Possible solutions include: CB1 neutral antagonists, peripheral CB1 neutral antagonists or inverse agonists, and CB1 negative allosteric modulators.

In the case of many disorders, targeting CB2, GPR18, GPR55, GPR119 or TRPV1 instead of CB1 may be a valid strategy as well. However, these proteins are not yet as well understood as CB1. Therefore, targeting them comes with a risk of encountering unknown adverse effects. For example, one has to bear in mind the possible pro-seizure activity of FAAH inhibitors and TRPV1 agonists [93,94], although more research is needed here. This shows that the understanding of each protein of ECS is needed in order to rationally target them. More research is needed here. Nevertheless, we gather the available information regarding potential therapeutic indications for each target, along with adverse effects. These data are summarized in a simplified way for the main proteins in Table 3 and in detail in Supplementary Table S2.

Moreover, it is important to emphasize that ECS, as a complex biological system, does not act alone, but rather in crosstalk and sometimes with important interactions with other systems. The often mentioned here TRPV1 is a protein of endovanilloid system, although, due to its activation by AEA, it may be as well regarded as a member of ECS. Other notable examples of close cooperation include: prostanoid [198], dopaminergic [351], glutaminergic [352], GABAergic [353], serotoninergic [354], opioid [355], and noradrenergic [356] systems. These interactions may be an effect of forming heterodimers of two GPCRs from ECS and other system [351] or crossing metabolic pathways of their endogenous ligands [198]. While targeting ECS presents a great potential, it is important to consider full picture and take into account complex biological factors and understand the physiological environment of this system. Such approach will increase the chances of successfully targeting ECS in drug design.

## Figures and Tables

**Table 1:** Potential ECS-mediated ways to treat nervous system-related disorders.

Protein	Ligand Type	Remarks	Evidence	References
**Pain**				
CB1	Agonist	Preferable CB1 peripheral agonists or CB1 PAMs	Well grounded	[78,79]
CB2	Agonist	In addition, CB2 PAMs		[80,81]
MAGL	Inhibitor			[82,84]
FAAH	Inhibitor			[83]
TRPV1	Antagonist			[159]
AEA reuptake proteins	Inhibitor			[85]
**Seizures**				
CB1	Agonist		Well grounded	[91]
MAGL	Inhibitor			[92]
TRPV1	Antagonist			[92,93]
ABHD6	Inhibitor			[92]
AEA reuptake proteins	Inhibitor			[92]
TRPV1	Agonist		Limited evidence	[98]
**Anxiety**				
CB1	Agonist		Well grounded	[111,115,118]
CB2	Agonist			[111,118]
MAGL	Inhibitor			[111]
FAAH	Inhibitor			[115,116]
TRPV1	Agonist			[118]
ine CB1	Antagonist	CB1 in lateral habenula	Limited evidence	[119]
FAAH	Enhancer	FAAH in basolateral complex of amygdala		[117]
**Depression**				
CB1	Agonist		Well grounded	[121]
MAGL	Inhibitor			[160]
FAAH	Inhibitor			[121,126]
CB1	Antagonist	Short-term	Limited evidence	[123,124]
CB2	Agonist			[121]
CB2	Antagonist			[123]
**Addiction**				
CB1	Antagonist	Preferable neutral antagonist or peripheral antagonist/inverse agonist	Well grounded	[129,130]
CB2	Agonist			[141,143]
ine CB1	Agonist	CB1 in insula; systemic in withdrawal syndrome	Limited evidence	[134,136]
CB2	Antagonist			[143]
MAGL	Inhibitor	MAGL in insula		[136]
**Cognitive functions**				
CB1	Agonist		Very complex topic, more reasearch needed	[154,157]
CB1	Antagonist			[146,147,148]
CB2	Agonist			[156,157]
MAGL	Inhibitor			[156]
FAAH	Inhibitor			[158]

**Table 2:** Approved drugs and compounds in clinical trials.

Name of the Active Ingredient	Mechanism of Action	Indications	Status	Remarks	References Clinical Trial IDs
Dronabinol (THC)	CB1 and CB2 partial agonist	Nausea and emesis associated with cancer chemotherapy Anorexia associated with weight loss in AIDS	FDA approved	Problems with therapeutic window	[309,327,328,329]
Nabilone	CB1 and CB2 partial agonist	Nausea and emesis associated with cancer chemotherapy	FDA approved	Problems with therapeutic window	[330,331]
CBD	CB1 negative allosteric modulator, TRPV1 agonist, GPR55 antagonist, 5-HT1A agonist, interacts with multiple other proteins	Lennox-Gastaut syndrome and Dravet syndrome (forms of epilepsy)	FDA approved		[40,100,101,102,103]
THC + CBD	See: dronabinol and CBD	Spasticity and pain in multiple sclerosis Cancer pain	Approved in multiple countries		[335,336]
Rimonabant	CB1 inverse agonist	Obesity	Withdrawn	Psychiatric adverse effects including depression and anxiety; subsequent suicides	[10]
JBT-101 (Lenabasum)	CB2 agonist	Diffuse cutaneous systemic sclerosis Dermatomyositis	Phase III trials		[338] NCT03398837 NCT03813160
		Cystic fibrosis Systemic sclerosis Systemic lupus erythematosus	Phase II trials		[349,350] NCT02465450 NCT02465437 NCT04043455
APD371 (Olorinab)	Peripheral CB2 agonist	Abdominal pain in Crohn’s disease Irritable bowel syndrome	Phase II trials		[339] NCT03155945 NCT04043455
NEO1940 (ART27.13)	Peripheral CB1 and CB2 agonist	Cancer related anorexia	Phase II trial planned	Phase I trials showed desired weight gain	[340]
CRB-4001	Peripheral CB1 inverse agonist	Nonalcoholic steatohepatitis	Phase I trials planned		[341,342]
ABX-1431	MAGL inhibitor	Tourette syndrome Chronic motor tic disorder	Phase II trial		[343] NCT03625453
NEO6860	TRPV1 antagonist	Osteoarthritis pain	Phase II trial	Phase I trials showed analgesic activity without hyperthermia; not effective in phase II trial	[159,347] NCT02712957 NCT02337543

**Table 3:** Potential indications for activation or inhibition of the main proteins of ECS.

Protein	Ligand Type	Indication	Risks	References
CB1	Agonist	Pain	Addiction Cognitive impairment Weight gain Erectile dysfunction	[78,79]
		Seizures		[91]
		Anxiety		[111,115,118]
		Depression		[121]
		Withdrawal syndrome		[134]
		Neurodegenerative disorders		[165,172,178]
		Spasticity in multiple sclerosis		[336]
		Hypertension		[246,247]
		Cancer		[269,270]
		Asthma		[296]
		Emesis and nausea		[308,309]
		Anorexia and weight loss		[327]
		Duchenne muscular dystrophy		[325]
	Antagonist	Addiction	Anxiety Depression Nausea	[129,130]
		Cognitive impairment		[146,147,148]
		Systemic sclerosis		[211]
		Pulmonary fibrosis		[212]
		Obesity		[215,216,218,219,221]
		Diabetes		[230,231]
		Nonalcoholic steatohepatitis		[243]
		Atherosclerosis		[257]
CB2	Agonist	Pain		[80,81]
		Anxiety		[111,118]
		Addiction		[141,143]
		Neurodegenerative disorders		[165]
		Inflammation		[192,196,208]
		Rheumatoid arthritis		[192]
		Atherosclerosis		[255]
		Systemic sclerosis		[210,211]
		Obesity		[77]
		Diabetes		[234,235]
		Cancer		[268,269]
		Inflammatory bowel disease		[305]
		Emesis and nausea		[308,310]
		Osteoporosis		[44]
	Antagonist	Immunoparalysis		[197]
		Renal fibrosis		[213]
MAGL	Inhibitor	Pain		[82,84]
		Seizures		[92]
		Tourette syndrome		[343]
		Anxiety		[111]
		Depression		[160]
		Cognitive impairment		[156]
		Neurodegenerative disorders		[176,183]
		Cancer		[279,281,282,283]
		Inflammatory bowel disease		[305]
FAAH	Inhibitor	Pain	Seizures Neurological disorder Disbalance in the kidney redox system Disbalance in phospholipid metabolism	[83]
		Anxiety		[115,116]
		Depression		[121,126]
		Cognitive impairment		[158]
		Neurodegenerative disorders		[179]
		Inflammation		[168,204,205]
		Hypertension		[250,251]
		Cancer		[285,286]
		Inflammatory bowel disease		[305]
TRPV1	Agonist	Anxiety	Seizures Aggravating pulmonary arterial hypertension	[118]
		Neurodegenerative disorders		[177]
		Hypertension		[253,254]
		Cancer		[275,285]
		Emesis and nausea		[310]
		Osteoporosis		[44]
	Antagonist	Pain	Hyperthermia	[84,159]
		Seizures		[92,93]

## Supplementary Materials

The following are available online at https://www.mdpi.com/1422-0067/21/8/2778/s1, Table S1: Diseases and disorders that could be treated by targeting ECS proteins, Table S2: Possible indications for activation or inhibition of the proteins of ECS.

## Abbreviations

The following abbreviations are used in this manuscript:

2-AG	2-arachidonoylglycerol
5-HT	5-hydroxytryptamine (serotonin)
5-HT1A	serotonin receptor 1A
5-HT2B	serotonin receptor 2B
5-HT3	serotonin receptor 3
Δ9-THC	Δ9-tetrahydrocannabinol
AA	arachidonic acid
ABHD6	α/β hydrolase domain 6
ABHD12	α/β hydrolase domain 12
AC	adenylyl cyclase
ACE	angiotensin-converting enzyme
AD	Alzheimer disease
AEA	N-arachidonoylethanolamine (anandamide)
AIDS	acquired immunodeficiency syndrome
ALS	amyotrophic lateral sclerosis
AM404	N-arachidonoylaminophenol
AMP	adenosine monophosphate
AMPA	α-amino-3-hydroxy-5-methyl-4-isoxazolepropionic acid
AMPK	AMP-activated protein kinase
AMT	anandamide membrane transporter
BLA	basolateral complex of amygdala
BP	blood pressure
cAMP	cyclic adenosine monophosphate
CB	cannabinoid
CB1	cannabinoid receptor type 1
CB2	cannabinoid receptor type 2
CBD	cannabidiol
CBDV	cannabidivarin
CBR	cannabinoid receptor
CIPN	chemotherapy-induced peripheral neuropathy
CNS	central nervous system
COX	cyclooxygenase
COX-2	cyclooxygenase 2
COX-3	cyclooxygenase 3
CXCR4	C-X-C chemokine receptor type 4
DA	dopamine
DAG	diacylglycerol
DAGL	diacylglycerol lipase
DN	diabetic neuropathy
DPP4	dipeptidyl peptidase 4
eCB	endocannabinoid
ECS	endocannabinoid system
ER	endoplasmic reticulum
ET	endotoxin tolerance
FAAH	fatty acid amide hydrolase
FABP	fatty-acid-binding protein
FCD	focal cortical dysplasia
FDA	Food and Drug Administration
GABA	γ-aminobutyric acid
GDP	guanosine diphosphate
GI	gastrointestinal
GIT	gastrointestinal tract
GLUT-2	glucose transporter 2
GPCR	G protein-coupled receptor
GPR18	G protein-coupled receptor 18
GPR55	G protein-coupled receptor 55
GPR119	G protein-coupled receptor 119
GTP	guanosine triphosphate
HAND	human immunodeficiency virus associated neurocognitive disorder
HER2	human epidermal growth factor receptor 2
HIV-1	human immunodeficiency virus 1
HPA	hypothalamic-pituitary-adrenal axis
HPO	hypothalamic-pituitary-ovarian axis
HSP70	70 kilodalton heat shock protein
IBD	inflammatory bowel disease
IL-1β	interleukin 1β
IL-6	interleukin 6
IL-18	interleukin 18
IMMA	indomethacin morpholinamide
iNOS	inducible nitric oxide synthase
IPAH	idiopathic pulmonary arterial hypertension
LHb	lateral habenula
LPI	lysophosphatidylinositol
LPS	lipopolysaccharide (endotoxin)
MAGL	monoacylglycerol lipase
MAPK	mitogen activated protein kinase
MCP-1	monocyte chemoattractant protein 1
MI	myocardial infraction
MOA	mechanism of action
mTORC1	mammalian target of rapamycin complex 1
NAAA	N-acylethanolamine acid amidase
NAFLD	nonalcoholic fatty liver disease
NAM	negative allosteric modulator
NAPE	N-acylphosphatidylethanolamine
NAPE-PLD	N-acylphosphatidylethanolamine-hydrolyzing phospholipase D
NASH	nonalcoholic steatohepatitis
NF-κB	nuclear factor-κB
NMDA	N-methyl-D-aspartate
NOS	nitric oxide synthase
NSAID	nonsteroidal anti-inflammatory drug
NSC	neural stem cell
OEA	oleoylethanolamine
OXPKOS	oxidative phosphorylation
PAM	positive allosteric modulator
PEA	palmitoylethanolamide
PG	prostaglandin
PGE2	prostaglandin E_2_
PPAR	peroxisome proliferator-activated receptor
PPARα	peroxisome proliferator-activated receptor α
PPARγ	peroxisome proliferator-activated receptor γ
PRCB	peripherally restricted cannabinoid
PTSD	post-traumatic stress disorder
RNA	ribonucleic acid
ROS	reactive oxygen species
SCA	spinocerebellar ataxia
SSc	systemic sclerosis
T2DM	type 2 diabetes mellitus
TBI	traumatic brain injury
THC	tetrahydrocannabinol
TIMP-1	tissue inhibitor of matrix metalloproteinases-1
TLR3	toll-like receptor 3
TLR4	toll-like receptor 4
TNF	tumor necrosis factor
TNF-α	tumor necrosis factor α
TRPV1	transient receptor potential vanilloid type 1 channel
WAT	white adipose tissue
