# Supplementary material for: A Guide to Targeting the Endocannabinoid System in Drug Design
Source: Int J Mol Sci. 2020 Apr 16;21(8):2778. doi: 10.3390/ijms21082778 (PMC7216112; doi:10.3390/ijms21082778)
Supplement: Supplementary file 1 [file ijms-21-02778-s001.pdf]

Supplementary Materials: A guide to targeting the endocannabinoid system in drug design

Adam Stasiulewicz, Katarzyna Znajdek, Monika Grudzień, Tomasz Pawiński and Joanna I. Sulkowska

Table S1. Diseases and disorders that could be treated by targeting ECS proteins.

Protein	Ligand type	Remarks	Evidence	References
		**Pain**		
CB1	Agonist	Preferable CB1 peripheral agonists or CB1 PAMs		[1,2]
CB2	Agonist	Also CB2 PAMs	Well grounded	[3,4]
TRPV1	Antagonist			[5]
FAAH	Inhibitor			[6]
MAGL	Inhibitor			[7,8]
AEA reuptake proteins	Inhibitor			[9]
		**Seizures**		
CB1	Agonist			[10]
MAGL	Inhibitor			[11]
AEA reuptake proteins	Inhibitor		Well grounded	[11]
ABHD6	Inhibitor			[11]
TRPV1	Antagonist			[11,12]
TRPV1	Agonist		Limited evidence	[13]
		**Anxiety**		
CB1	Agonist			[14–16]
CB2	Agonist			[15,16]
FAAH	Inhibitor		Well grounded	[14,17]
MAGL	Inhibitor			[16]
TRPV1	Agonist			[15]
FAAH	Enhancer	FAAH in basolateral complex of amygdala	Limited evidence	[18]
CB1	Antagonist	CB1 in lateral habenula		[19]
		**Depression**		
CB1	Agonist			[20]
FAAH	Inhibitor		Well grounded	[20,21]
MAGL	Inhibitor			[22]
CB2	Agonist			[20]
CB1	Antagonist	short-term	Limited evidence	[23,24]
CB2	Antagonist			[24]
		**Addiction**		
CB1	Antagonist	Preferable neutral antagonist or peripheral antagonist/inverse agonist	Well grounded	[25,26]
CB2	Agonist			[27,28]
CB1	Agonist	CB1 in insula; systemic in withdrawal syndrome		[29,30]
CB2	Antagonist		Limited evidence	[28]
MAGL	Inhibitor	MAGL in insula		[29]
		**Cognitive functions**		
FAAH	Inhibitor			[31]
MAGL	Inhibitor			[32]
CB1	Antagonist		Very complex topic, more reasearch needed	[33–35]

CB1	Agonist			[36,37]
CB2	Agonist			[32,37]
**Neurodegeneration**				
CB1	Agonist		Well grounded	[38–40]
MAGL	Inhibitor			[41,42]
FAAH	Inhibitor			[43]
CB2	Agonist			[38]
TRPV1	Agonist		Limited evidence	[44]
GPR55	Agonist			[40,45]
GPR55	Antagonist			[46]
CB1	Antagonist	Focal cortical dysplasia		[47]
**Inflammatory and autoimmune diseases**				
CB2	Agonist	Inflammatory diseases	Well grounded	[48–52]
CB2	Antagonist	Immunoparalysis, renal fibrosis		[53,54]
FAAH	Inhibitor			[55–57]
PPARγ	Agonist			[51,52]
CB1	Antagonist	Systemic sclerosis, pulmonary fibrosis		[51,58]
CB1	Agonist		Limited evidence	[59]
CB1	Antagonist			[60]
TRPV1	Agonist			[61]
TRPV1	Antagonist			[62]
GPR55	Agonist			[50]
GPR55	Antagonist			[46]
MAGL	Inhibitor			[63]
**Obesity**				
CB1	Antagonist	Preferable peripheral antagonist/inverse agonist	Well grounded	[64–68]
CB2	Agonist			[69]
GPR55	Agonist			[70,71]
GPR18	Agonist		Limited evidence	[71]
**Diabetes**				
CB1	Antagonist	Preferable peripheral antagonist/inverse agonist	Well grounded	[72,73]
CB2	Agonist			[74,75]
GPR119	Agonist			[76]
GPR55	Agonist		Limited evidence	[77,78]
**Hepatic diseases**				
CB1	Antagonist		Well grounded	[79,80]
CB2	Agonist			[81]
GPR119	Agonist		Limited evidence	[82]
**Hypertension**				
CB1	Agonist	Peripheral agonist	Well grounded	[83,84]
FAAH	Inhibitor			[85,86]
**Atherosclerosis**				
CB1	Antagonist		Well grounded	[87]
CB2	Agonist			[88]
MAGL	Inhibitor			[89]
GPR55	Agonist		Limited evidence	[90]
PPARα	Agonist			[90]
**Myocardial dysfunctions**				
CB2	Agonist	Deleterious effect in myocardial infraction	Limited evidence	[91,92]

TRPV1	Agonist			[83]
MAGL	Inhibitor			[93]
**Cancer**				
CB1	Agonist		Well grounded	[94,95]
CB2	Agonist			[94,96]
GPR55	Antagonist			[97–100]
TRPV1	Agonist			[101,102]
FAAH	Inhibitor			[102,103]
MAGL	Inhibitor			[104–107]
NAAA	Inhibitor		Limited evidence	[108]
**Respiratory disorders**				
CB1	Agonist		Well grounded	[109]
**Gastroenterology**				
CB1	Agonist	Emesis and nausea, anorexia, malnutrition	Well grounded	[110]
CB2	Agonist		Limited evidence	[110–112]
FAAH	Inhibitor			[112]
MAGL	Inhibitor			[112]
GPR55	Antagonist			[113]
**Osteology**				
CB1	Antagonist		Well grounded	[114,115]
CB2	Agonist		Limited evidence	[114]
TRPV1	Antagonist			[114]
**Reproductive system**				
CB1	Antagonist	Potential use in erectile dysfunctions, preferable peripheral antagonist/inverse agonist	Limited evidence	[116]
**Dermatology**				
CB1	Agonist	Anti-fibrotic effect, hair growth	Well grounded	[117,118]
CB1	Antagonist	Anti-inflammatory		[117,118]
CB2	Agonist	Anti-acne, anti-seborrhea effect		[117,118]
CB2	Antagonist	Anti-dryness, anti-inflammatory, anti-fibrotic effect		[117,118]
eCB reuptake proteins	Inhibitor	In conditions with inflammation and dryness	Limited evidence	[119]
**Genetic disorders**				
CB1	Antagonist	Duchenne muscular dystrophy	Limited evidence	[120]

Table S2. Possible indications for activation or inhibition of the proteins of ECS.

Protein	Ligand type	Indication	Risk	References
CB1	Agonist	Pain		[1,2]
		Seizures		[10]
		Anxiety		[14–16]
		Depression	Addiction	[20]
		Withdrawal syndrome	Cognitive impairment	[30]
		Neurodegenerative disorders	Weight gain	[38–40]
		Spasticity in multiple sclerosis	Erectile dysfunction	[121]
		Hypertension		[83,84]
		Cancer		[94,95]
		Asthma		[109]
		Emesis and nausea		[110,122]
		Anorexia and weight loss		[123]
		Duchenne muscular dystrophy		[120]
	Antagonist	Addiction		[25,26]
		Cognitive impairment		[33–35]
		Systemic sclerosis	Anxiety	[51]
		Pulmonary fibrosis	Depression	[58]
		Obesity	Nausea	[64–68]
		Diabetes		[72,73]
		Nonalcoholic steatohepatitis		[79]
		Atherosclerosis		[87]
CB2	Agonist	Pain		[3,4]
		Anxiety		[15,16]
		Addiction		[27,28]
		Neurodegenerative disorders		[38]
		Inflammation		[48–50]
		Rheumatoid arthritis		[48]
		Atherosclerosis		[88]
		Systemic sclerosis		[51,52]
		Obesity		[69]
		Diabetes		[74,75]
		Cancer		[94,96]
		Inflammatory bowel disease		[112]
		Emesis and nausea		[110,111]
		Osteoporosis		[114]
	Antagonist	Immunoparalysis		[54]
		Renal fibrosis		[53]
FAAH	Inhibitor	Pain		[6]
		Anxiety		[14,17]
		Depression	Seizures	[20,21]
		Cognitive impairment	Neurological disorder	[31]
		Neurodegenerative disorders	Disbalance in the kidney redox system	[43]
		Inflammation	Disbalance in phospholipid metabolism	[55–57]
		Hypertension		[85,86]
		Cancer		[102,103]
		Inflammatory bowel disease		[112]
MAGL	Inhibitor	Pain		[7,8]
		Seizures		[11]
		Tourette syndrome		[124]
		Anxiety		[16]
		Depression		[22]

		Cognitive impairment		[32]
		Neurodegenerative disorders		[41,42]
		Cancer		[104–107]
		Inflammatory bowel disease		[112]
TRPV1	Agonist	Anxiety	Seizures<br>Aggravating pulmonary arterial hypertension	[15]
		Neurodegenerative disorders		[44]
		Hypertension		[125,126]
		Cancer		[101,102]
		Emesis and nausea		[111]
		Osteoporosis		[114]
	Antagonist	Pain	Hyperthermia	[5,7]
		Seizures		[11,12]
PPARγ	Agonist	Systemic sclerosis		[51,52]
GPR18	Agonist	Obesity	Liver and kidney damage	[71]
GPR55	Agonist	Neurodegenerative disorders	Liver and kidney damage	[40,45]
		Inflammation		[50]
		Obesity		[70,71]
		Diabetes		[77,78]
		Atherosclerosis		[90]
	Antagonist	Neurodegenerative disorders		[46]
		Cancer		[97–100]
GPR119	Agonist	Diabetes		[76]
		Dyslipidemia		[82]
		Nonalcoholic steatohepatitis		[82]
ABHD6	Inhibitor	Seizures		[11]
AEA reuptake proteins	Inhibitor	Pain		[9]
		Seizures		[11]
		Skin inflammation		[119]

Abbreviations

The following abbreviations are used in the Supplementary Materials:

ABHD6	α/β hydrolase domain 6
AEA	N-arachidonoylethanolamine (anandamide)
CB1	cannabinoid receptor type 1
CB2	cannabinoid receptor type 2
eCB	endocannabinoid
FAAH	fatty acid amide hydrolase
GPR18	G protein-coupled receptor 18
GPR55	G protein-coupled receptor 55
GPR119	G protein-coupled receptor 119
MAGL	monoacylglycerol lipase
NAAA	N-acylethanolamine acid amidase
PAM	positive allosteric modulator
PPARα	peroxisome proliferator-activated receptor α
PPARγ	peroxisome proliferator-activated receptor γ
TRPV1	transient receptor potential vanilloid type 1 channel

References

1. Mulpuri, Y.; Marty, V.N.; Munier, J.J.; Mackie, K.; Schmidt, B.L.; Seltzman, H.H.; Spigelman, I. Synthetic peripherally-restricted cannabinoid suppresses chemotherapy-induced peripheral neuropathy pain symptoms by CB1 receptor activation. *Neuropharmacology* **2018**, *139*, 85–97.
2. Slivicki, R.A.; Xu, Z.; Kulkarni, P.M.; Pertwee, R.G.; Mackie, K.; Thakur, G.A.; Hohmann, A.G. Positive allosteric modulation of cannabinoid receptor type 1 suppresses pathological pain without producing tolerance or dependence. *Biological Psychiatry* **2018**, *84*, 722–733.
3. Gado, F.; Di Cesare Mannelli, L.; Lucarini, E.; Bertini, S.; Cappelli, E.; Digiacomo, M.; Stevenson, L.A.; Macchia, M.; Tuccinardi, T.; Ghelardini, C.; others. Identification of the first synthetic allosteric modulator of the CB2 receptors and evidence of its efficacy for neuropathic pain relief. *Journal of Medicinal Chemistry* **2019**, *62*, 276–287.
4. Nent, E.; Nozaki, C.; Schmöle, A.C.; Otte, D.; Zimmer, A. CB2 receptor deletion on myeloid cells enhanced mechanical allodynia in a mouse model of neuropathic pain. *Scientific Reports* **2019**, *9*, 7468.
5. Brown, W.; Leff, R.L.; Griffin, A.; Hossack, S.; Aubray, R.; Walker, P.; Chiche, D.A. Safety, pharmacokinetics, and pharmacodynamics study in healthy subjects of oral NEO6860, a modality selective transient receptor potential vanilloid subtype 1 antagonist. *The Journal of Pain* **2017**, *18*, 726–738.
6. Brindisi, M.; Borrelli, G.; Brogi, S.; Grillo, A.; Maramai, S.; Paolino, M.; Benedusi, M.; Pecorelli, A.; Valacchi, G.; Di Cesare Mannelli, L.; others. Development of Potent Inhibitors of Fatty Acid Amide Hydrolase Useful for the Treatment of Neuropathic Pain. *ChemMedChem* **2018**, *13*, 2090–2103.
7. Malek, N.; Starowicz, K. Dual-acting compounds targeting endocannabinoid and endovanilloid systems—a novel treatment option for chronic pain management. *Frontiers in Pharmacology* **2016**, *7*, 257.
8. Clapper, J.R.; Henry, C.L.; Niphakis, M.J.; Knize, A.M.; Coppola, A.R.; Simon, G.M.; Ngo, N.; Herbst, R.A.; Herbst, D.M.; Reed, A.W.; others. Monoacylglycerol lipase inhibition in human and rodent systems supports clinical evaluation of endocannabinoid modulators. *Journal of Pharmacology and Experimental Therapeutics* **2018**, *367*, 494–508.
9. da Fonseca Pacheco, D.; Romero, T.R.L.; Duarte, I.D.G. Ketamine induces central antinociception mediated by endogenous cannabinoids and activation of CB1 receptors. *Neuroscience Letters* **2019**, *699*, 140–144.
10. Colangeli, R.; Di Maio, R.; Pierucci, M.; Deidda, G.; Casarrubea, M.; Di Giovanni, G. Synergistic action of CB1 and 5-HT2B receptors in preventing pilocarpine-induced status epilepticus in rats. *Neurobiology of Disease* **2019**, *125*, 135–145.
11. Zareie, P.; Sadegh, M.; Palizvan, M.R.; Moradi-Chameh, H. Anticonvulsive effects of endocannabinoids; an investigation to determine the role of regulatory components of endocannabinoid metabolism in the Pentylenetetrazol induced tonic-clonic seizures. *Metabolic Brain Disease* **2018**, *33*, 939–948.
12. Manna, S.S.; Umathe, S.N. Involvement of transient receptor potential vanilloid type 1 channels in the pro-convulsant effect of anandamide in pentylenetetrazole-induced seizures. *Epilepsy Research* **2012**, *100*, 113–124.
13. Suemaru, K.; Yoshikawa, M.; Aso, H.; Watanabe, M. TRPV1 mediates the anticonvulsant effects of acetaminophen in mice. *Epilepsy Research* **2018**, *145*, 153–159.
14. Danandeh, A.; Vozella, V.; Lim, J.; Oveisi, F.; Ramirez, G.L.; Mears, D.; Wynn, G.; Piomelli, D. Effects of fatty acid amide hydrolase inhibitor URB597 in a rat model of trauma-induced long-term anxiety. *Psychopharmacology* **2018**, *235*, 3211–3221.
15. Surkin, P.N.; Gallino, S.L.; Luce, V.; Correa, F.; Fernandez-Solari, J.; De Laurentiis, A. Pharmacological augmentation of endocannabinoid signaling reduces the neuroendocrine response to stress. *Psychoneuroendocrinology* **2018**, *87*, 131–140.
16. Viana, T.G.; Bastos, J.R.; Costa, R.B.; Hott, S.C.; Mansur, F.S.; Coimbra, C.C.; Resstel, L.B.; Aguiar, D.C.; Moreira, F.A. Hypothalamic endocannabinoid signalling modulates aversive responses related to panic attacks. *Neuropharmacology* **2019**, *148*, 284–290.
17. Mayo, L.M.; Asratian, A.; Lindé, J.; Morena, M.; Haataja, R.; Hammar, V.; Augier, G.; Hill, M.N.; Heilig, M. Elevated anandamide, enhanced recall of fear extinction, and attenuated stress responses following inhibition of fatty acid amide hydrolase (FAAH): a randomized, controlled experimental medicine trial. *Biological Psychiatry* **2019**, *87*, 538–547.

18. Morena, M.; Aukema, R.J.; Leitl, K.D.; Rashid, A.J.; Vecchiarelli, H.A.; Josselyn, S.A.; Hill, M.N. Upregulation of Anandamide Hydrolysis in the Basolateral Complex of Amygdala Reduces Fear Memory Expression and Indices of Stress and Anxiety. *Journal of Neuroscience* **2019**, *39*, 1275–1292.
19. Berger, A.L.; Henricks, A.M.; Lugo, J.M.; Wright, H.R.; Warrick, C.R.; Sticht, M.A.; Morena, M.; Bonilla, I.; Laredo, S.A.; Craft, R.M.; others. The lateral habenula directs coping styles under conditions of stress via recruitment of the endocannabinoid system. *Biological Psychiatry* **2018**, *84*, 611–623.
20. Burstein, O.; Shoshan, N.; Doron, R.; Akirav, I. Cannabinoids prevent depressive-like symptoms and alterations in BDNF expression in a rat model of PTSD. *Progress in Neuro-psychopharmacology and Biological Psychiatry* **2018**, *84*, 129–139.
21. Wang, Y.; Zhang, X. FAAH inhibition produces antidepressant-like efforts of mice to acute stress via synaptic long-term depression. *Behavioural Brain Research* **2017**, *324*, 138–145.
22. Zhong, P.; Wang, W.; Pan, B.; Liu, X.; Zhang, Z.; Long, J.Z.; Zhang, H.t.; Cravatt, B.F.; Liu, Q.s. Monoacylglycerol lipase inhibition blocks chronic stress-induced depressive-like behaviors via activation of mTOR signaling. *Neuropsychopharmacology* **2014**, *39*, 1763–1776.
23. Shearman, L.; Rosko, K.; Fleischer, R.; Wang, J.; Xu, S.; Tong, X.; Rocha, B.A. Antidepressant-like and anorectic effects of the cannabinoid CB1 receptor inverse agonist AM251 in mice. *Behavioural Pharmacology* **2003**, *14*, 573–582.
24. Khakpai, F.; Ebrahimi-Ghiri, M.; Alijanpour, S.; Zarrindast, M.R. Ketamine-induced antidepressant like effects in mice: A possible involvement of cannabinoid system. *Biomedicine & Pharmacotherapy* **2019**, *112*, 108717.
25. Balla, A.; Dong, B.; Shilpa, B.M.; Vemuri, K.; Makriyannis, A.; Pandey, S.C.; Sershen, H.; Suckow, R.F.; Vinod, K.Y. Cannabinoid-1 receptor neutral antagonist reduces binge-like alcohol consumption and alcohol-induced accumbal dopaminergic signaling. *Neuropharmacology* **2018**, *131*, 200–208.
26. He, X.h.; Jordan, C.J.; Vemuri, K.; Bi, G.h.; Zhan, J.; Gardner, E.L.; Makriyannis, A.; Wang, Y.l.; Xi, Z.x. Cannabinoid CB 1 receptor neutral antagonist AM4113 inhibits heroin self-administration without depressive side effects in rats. *Acta Pharmacologica Sinica* **2019**, *40*, 365–373.
27. Navarrete, F.; García-Gutiérrez, M.S.; Manzanares, J. Pharmacological regulation of cannabinoid CB2 receptor modulates the reinforcing and motivational actions of ethanol. *Biochemical Pharmacology* **2018**, *157*, 227–234.
28. Martín-Sánchez, A.; Warnault, V.; Montagud-Romero, S.; Pastor, A.; Mondragón, N.; De La Torre, R.; Valverde, O. Alcohol-induced conditioned place preference is modulated by CB2 cannabinoid receptors and modifies levels of endocannabinoids in the mesocorticolimbic system. *Pharmacology Biochemistry and Behavior* **2019**, *183*, 22–31.
29. Centanni, S.W.; Morris, B.D.; Luchsinger, J.R.; Bedse, G.; Fetterly, T.L.; Patel, S.; Winder, D.G. Endocannabinoid control of the insular-bed nucleus of the stria terminalis circuit regulates negative affective behavior associated with alcohol abstinence. *Neuropsychopharmacology* **2019**, *44*, 526–537.
30. Ferreira, J.V.; Chaves, G.A.; Marino, B.L.; Sousa, K.P.; Souza, L.R.; Brito, M.F.; Teixeira, H.R.; da Silva, C.H.; Santos, C.B.; Hage-Melim, L.I. Cannabinoid type 1 receptor (CB1) ligands with therapeutic potential for withdrawal syndrome in chemical dependents of Cannabis sativa. *ChemMedChem* **2017**, *12*, 1408–1416.
31. Contarini, G.; Ferretti, V.; Papaleo, F. Acute administration of URB597 fatty acid amide hydrolase (FAAH) inhibitor prevents attentional impairments by distractors in adolescent mice. *Frontiers in Pharmacology* **2019**, *10*, 787.
32. Ratano, P.; Petrella, C.; Forti, F.; Passeri, P.P.; Morena, M.; Palmery, M.; Trezza, V.; Severini, C.; Campolongo, P. Pharmacological inhibition of 2-arachidonoilglycerol hydrolysis enhances memory consolidation in rats through CB2 receptor activation and mTOR signaling modulation. *Neuropharmacology* **2018**, *138*, 210–218.
33. Rabbani, M.; Vaseghi, G.; Hajhashemi, V. AM281, Cannabinoid Antagonist/Inverse agonist, ameliorates scopolamine-induced cognitive deficit. *Iranian Journal of Basic Medical Sciences* **2012**, *15*, 1106–1110.
34. Xu, X.; Jiang, S.; Xu, E.; Wu, X.; Zhao, R. Inhibition of CB1 receptor ameliorates spatial learning and memory impairment in mice with traumatic brain injury. *Neuroscience Letters* **2019**, *696*, 127–131.
35. Navarro-Romero, A.; Vázquez-Oliver, A.; Gomis-González, M.; Garzón-Montesinos, C.; Falcón-Moya, R.; Pastor, A.; Martín-García, E.; Pizarro, N.; Busquets-Garcia, A.; Revest, J.M.; others. Cannabinoid type-1 receptor blockade restores neurological phenotypes in two models for Down syndrome. *Neurobiology of Disease* **2019**, *125*, 92–106.

36. Morena, M.; Roozendaal, B.; Trezza, V.; Ratano, P.; Peloso, A.; Hauer, D.; Atsak, P.; Trabace, L.; Cuomo, V.; McGaugh, J.L.; others. Endogenous cannabinoid release within prefrontal-limbic pathways affects memory consolidation of emotional training. *Proceedings of the National Academy of Sciences* **2014**, *111*, 18333–18338.
37. Rivera, P.; del Mar Fernández-Arjona, M.; Silva-Peña, D.; Blanco, E.; Vargas, A.; López-Ávalos, M.D.; Grondona, J.M.; Serrano, A.; Pavón, F.J.; de Fonseca, F.R.; others. Pharmacological blockade of fatty acid amide hydrolase (FAAH) by URB597 improves memory and changes the phenotype of hippocampal microglia despite ethanol exposure. *Biochemical Pharmacology* **2018**, *157*, 244–257.
38. Sanchez-Rodriguez, M.A.; Gomez, O.; Esteban, P.F.; Garcia-Ovejero, D.; Molina-Holgado, E. The endocannabinoid 2-arachidonoylglycerol regulates oligodendrocyte progenitor cell migration. *Biochemical Pharmacology* **2018**, *157*, 180–188.
39. Crunfli, F.; Vrechi, T.A.; Costa, A.P.; Torrão, A.S. Cannabinoid Receptor Type 1 Agonist ACEA Improves Cognitive Deficit on STZ-Induced Neurotoxicity Through Apoptosis Pathway and NO Modulation. *Neurotoxicity Research* **2019**, *35*, 516–529.
40. Martínez-Pinilla, E.; Aguinaga, D.; Navarro, G.; Rico, A.J.; Oyarzábal, J.; Sánchez-Arias, J.A.; Lanciego, J.L.; Franco, R. Targeting CB 1 and GPR55 Endocannabinoid Receptors as a Potential Neuroprotective Approach for Parkinson's Disease. *Molecular Neurobiology* **2019**, *56*, 5900–5910.
41. Covey, D.P.; Dantrassy, H.M.; Yohn, S.E.; Castro, A.; Conn, P.J.; Mateo, Y.; Cheer, J.F. Inhibition of endocannabinoid degradation rectifies motivational and dopaminergic deficits in the Q175 mouse model of Huntington's disease. *Neuropsychopharmacology* **2018**, *43*, 2056—-2063.
42. Chen, L.; Yan, Y.; Chen, T.; Zhang, L.; Gao, X.; Du, C.; Du, H. Forsythiaside prevents β-amyloid-induced hippocampal slice injury by upregulating 2-arachidonoylglycerol via cannabinoid receptor 1-dependent NF-κB pathway. *Neurochemistry International* **2019**, *125*, 57–66.
43. Viveros-Paredes, J.; Gonzalez-Castañeda, R.; Escalante-Castañeda, A.; Tejeda-Martínez, A.; Castañeda-Achutiguí, F.; Flores-Soto, M. Effect of inhibition of fatty acid amide hydrolase on MPTP-induced dopaminergic neuronal damage. *Neurología (English Edition)* **2019**, *34*, 143–152.
44. Balleza-Tapia, H.; Crux, S.; Andrade-Talavera, Y.; Dolz-Gaiton, P.; Papadia, D.; Chen, G.; Johansson, J.; Fisahn, A. TrpV1 receptor activation rescues neuronal function and network gamma oscillations from Aβ-induced impairment in mouse hippocampus in vitro. *eLife* **2018**, *7*, e37703.
45. Hill, J.D.; Zuluaga-Ramirez, V.; Gajghate, S.; Winfield, M.; Persidsky, Y. Activation of GPR55 increases neural stem cell proliferation and promotes early adult hippocampal neurogenesis. *British Journal of Pharmacology* **2018**, *175*, 3407–3421.
46. Saliba, S.W.; Jauch, H.; Gargouri, B.; Keil, A.; Hurrle, T.; Volz, N.; Mohr, F.; van der Stelt, M.; Bräse, S.; Fiebich, B.L. Anti-neuroinflammatory effects of GPR55 antagonists in LPS-activated primary microglial cells. *Journal of Neuroinflammation* **2018**, *15*, 322.
47. García-Rincón, D.; Díaz-Alonso, J.; Ortega, Z.; de Salas-Quiroga, A.; Paraíso-Luna, J.; Aguareles, J.; Jou, C.; De Prada, I.; Martinez Cerdeño, V.; Aronica, E.; others. Contribution of altered endocannabinoid system to overactive mTORC1 signalling in focal cortical dysplasia. *Frontiers in Pharmacology* **2018**, *9*, 1508.
48. Turcotte, C.; Blanchet, M.R.; Laviolette, M.; Flamand, N. The CB 2 receptor and its role as a regulator of inflammation. *Cellular and Molecular Life Sciences* **2016**, *73*, 4449–4470.
49. Toguri, J.; Leishman, E.; Szczesniak, A.; Laprairie, R.; Oehler, O.; Straiker, A.; Kelly, M.; Bradshaw, H. Inflammation and CB2 signaling drive novel changes in the ocular lipidome and regulate immune cell activity in the eye. *Prostaglandins & Other Lipid Mediators* **2018**, *139*, 54–62.
50. Cruz, S.L.; Sánchez-Miranda, E.; Castillo-Arellano, J.I.; Cervantes-Villagrana, R.D.; Ibarra-Sánchez, A.; González-Espinosa, C. Anandamide inhibits FcεRI-dependent degranulation and cytokine synthesis in mast cells through CB2 and GPR55 receptor activation. Possible involvement of CB2-GPR55 heteromers. *International Immunopharmacology* **2018**, *64*, 298–307.
51. del Rio, C.; Cantarero, I.; Palomares, B.; Gómez-Cañas, M.; Fernández-Ruiz, J.; Pavicic, C.; García-Martín, A.; Luz Bellido, M.; Ortega-Castro, R.; Pérez-Sánchez, C.; others. VCE-004.3, a cannabidiol aminoquinone derivative, prevents bleomycin-induced skin fibrosis and inflammation through PPARγ-and CB2 receptor-dependent pathways. *British Journal of Pharmacology* **2018**, *175*, 3813–3831.
52. García-Martín, A.; Garrido-Rodríguez, M.; Navarrete, C.; Caprioglio, D.; Palomares, B.; DeMesa, J.; Rollland, A.; Appendino, G.; Muñoz, E. Cannabinoid derivatives acting as dual PPARγ/CB2 agonists as therapeutic agents for systemic sclerosis. *Biochemical Pharmacology* **2019**, *163*, 321–334.

53. Zhou, L.; Zhou, S.; Yang, P.; Tian, Y.; Feng, Z.; Xie, X.Q.; Liu, Y. Targeted inhibition of the type 2 cannabinoid receptor is a novel approach to reduce renal fibrosis. *Kidney International* **2018**, *94*, 756–772.
54. Espinosa-Riquer, Z.P.; Ibarra-Sánchez, A.; Vibhushan, S.; Bratti, M.; Charles, N.; Blank, U.; Rodríguez-Manzo, G.; González-Espinosa, C. TLR4 Receptor Induces 2-AG–Dependent Tolerance to Lipopolysaccharide and Trafficking of CB2 Receptor in Mast Cells. *The Journal of Immunology* **2019**, *202*, 2360–2371.
55. Chen, H.J.C.; Spiers, J.G.; Sernia, C.; Lavidis, N.A. Inhibition of fatty acid amide hydrolase by PF-3845 alleviates the nitrergic and proinflammatory response in rat hippocampus following acute stress. *International Journal of Neuropsychopharmacology* **2018**, *21*, 786–795.
56. Flannery, L.E.; Kerr, D.M.; Finn, D.P.; Roche, M. FAAH inhibition attenuates TLR3-mediated hyperthermia, nociceptive-and anxiety-like behaviour in female rats. *Behavioural Brain Research* **2018**, *353*, 11–20.
57. Tanaka, M.; Yagyu, K.; Sackett, S.; Zhang, Y. Anti-Inflammatory Effects by Pharmacological Inhibition or Knockdown of Fatty Acid Amide Hydrolase in BV2 Microglial Cells. *Cells* **2019**, *8*, 491.
58. Cinar, R.; Gochuico, B.R.; Iyer, M.R.; Jourdan, T.; Yokoyama, T.; Park, J.K.; Coffey, N.J.; Pri-Chen, H.; Szanda, G.; Liu, Z.; others. Cannabinoid CB1 receptor overactivity contributes to the pathogenesis of idiopathic pulmonary fibrosis. *JCI Insight* **2017**, *2*, 92281.
59. Nam, G.; Jeong, S.K.; Park, B.M.; Lee, S.H.; Kim, H.J.; Hong, S.P.; Kim, B.; Kim, B.W. Selective cannabinoid receptor-1 agonists regulate mast cell activation in an oxazolone-induced atopic dermatitis model. *Annals of Dermatology* **2016**, *28*, 22–29.
60. Ambrożewicz, E.; Wójcik, P.; Wroński, A.; Łuczaj, W.; Jastrząb, A.; Žarković, N.; Skrzydlewska, E. Pathophysiological Alterations of Redox Signaling and Endocannabinoid System in Granulocytes and Plasma of Psoriatic Patients. *Cells* **2018**, *7*, 159.
61. Tsuji, F.; Aono, H. Role of transient receptor potential vanilloid 1 in inflammation and autoimmune diseases. *Pharmaceuticals* **2012**, *5*, 837–852.
62. Kim, J.H. The Emerging Role of TRPV1 in Airway Inflammation. *Allergy, Asthma & Immunology Research* **2018**, *10*, 187–188.
63. Rahmani, M.R.; Shamsizadeh, A.; Moghadam-Ahmadi, A.; Bazmandegan, G.; Allahtavakoli, M. JZL184, as a monoacylglycerol lipase inhibitor, down-regulates inflammation in a cannabinoid pathway dependent manner. *Biomedicine & Pharmacotherapy* **2018**, *103*, 1720–1726.
64. Chorvat, R.J. Peripherally restricted CB1 receptor blockers. *Bioorganic & Medicinal Chemistry Letters* **2013**, *23*, 4751–4760.
65. Seltzman, H.H.; Maitra, R.; Bortoff, K.; Henson, J.; Reggio, P.H.; Wesley, D.; Tam, J. Metabolic profiling of CB1 neutral antagonists. *Methods in Enzymology* **2017**, *593*, 199–215.
66. Han, J.H.; Shin, H.; Park, J.Y.; Rho, J.G.; Son, D.H.; Kim, K.W.; Seong, J.K.; Yoon, S.H.; Kim, W. A novel peripheral cannabinoid 1 receptor antagonist, AJ5012, improves metabolic outcomes and suppresses adipose tissue inflammation in obese mice. *The FASEB Journal* **2019**, *33*, 4314–4326.
67. de Azua, I.R.; Lutz, B. Multiple endocannabinoid-mediated mechanisms in the regulation of energy homeostasis in brain and peripheral tissues. *Cellular and Molecular Life Sciences* **2019**, *76*, 1341–1363.
68. Ogden, S.B.; Malamas, M.S.; Makriyannis, A.; Eckel, L.A. The novel cannabinoid 1 receptor agonist AM11101 increases food intake in female rats. *British Journal of Pharmacology* **2019**, *176*, 3972–3982.
69. Parsons, L.H.; Hurd, Y.L. Endocannabinoid signalling in reward and addiction. *Nature Reviews Neuroscience* **2015**, *16*, 579–594.
70. Lipina, C.; Walsh, S.K.; Mitchell, S.E.; Speakman, J.R.; Wainwright, C.L.; Hundal, H.S. GPR55 deficiency is associated with increased adiposity and impaired insulin signaling in peripheral metabolic tissues. *The FASEB Journal* **2019**, *33*, 1299–1312.
71. Simcocks, A.; Jenkin, K.; O'keefe, L.; Samuel, C.; Mathai, M.; McAinch, A.; Hryciw, D. Atypical cannabinoid ligands O-1602 and O-1918 administered chronically in diet-induced obesity. *Endocrine Connections* **2019**, *8*, 203—-216.
72. Hinden, L.; Udi, S.; Drori, A.; Gammal, A.; Nemirovski, A.; Hadar, R.; Baraghithy, S.; Permyakova, A.; Geron, M.; Cohen, M.; others. Modulation of renal GLUT2 by the cannabinoid-1 receptor: implications for the treatment of diabetic nephropathy. *Journal of the American Society of Nephrology* **2018**, *29*, 434–448.
73. Hinden, L.; Tam, J. Do Endocannabinoids Regulate Glucose Reabsorption in the Kidney? *Nephron* **2019**, *143*, 24—-27.

74. Wu, A.; Hu, P.; Lin, J.; Xia, W.; Zhang, R. Activating cannabinoid receptor 2 protects against diabetic cardiomyopathy through autophagy induction. *Frontiers in Pharmacology* **2018**, *9*, 1292.
75. Kumawat, V.S.; Kaur, G. Therapeutic potential of cannabinoid receptor 2 in the treatment of diabetes mellitus and its complications. *European Journal of Pharmacology* **2019**, *862*, 172628.
76. Huan, Y.; Jiang, Q.; Li, G.; Bai, G.; Zhou, T.; Liu, S.; Li, C.; Liu, Q.; Sun, S.; Yang, M.; others. The dual DPP4 inhibitor and GPR119 agonist HBK001 regulates glycemic control and beta cell function ex and in vivo. *Scientific Reports* **2017**, *7*, 4351.
77. González-Mariscal, I.; Egan, J.M. Endocannabinoids in the Islets of Langerhans: the ugly, the bad, and the good facts. *American Journal of Physiology-Endocrinology and Metabolism* **2018**, *315*, E174–E179.
78. Vong, C.T.; Tseng, H.H.L.; Kwan, Y.W.; Lee, S.M.Y.; Hoi, M.P.M. Novel protective effect of O-1602 and abnormal cannabidiol, GPR55 agonists, on ER stress-induced apoptosis in pancreatic β-cells. *Biomedicine & Pharmacotherapy* **2019**, *111*, 1176–1186.
79. Chang, E.; Kim, D.H.; Yang, H.; Lee, D.H.; Bae, S.H.; Park, C.Y. CB1 receptor blockade ameliorates hepatic fat infiltration and inflammation and increases Nrf2-AMPK pathway in a rat model of severely uncontrolled diabetes. *PloS One* **2018**, *13*, e0206152.
80. Jorgačević, B.; Vučević, D.; Vesković, M.; Mladenović, D.; Vukićević, D.; Vukićević, R.J.; Todorović, V.; Radosavljević, T. The effect of cannabinoid receptor 1 blockade on adipokine and proinflammatory cytokine concentration in adipose and hepatic tissue in mice with nonalcoholic fatty liver disease. *Canadian Journal of Physiology and Pharmacology* **2019**, *97*, 120–129.
81. Bazwinsky-Wutschke, I.; Zipprich, A.; Dehghani, F. Endocannabinoid System in Hepatic Glucose Metabolism, Fatty Liver Disease, and Cirrhosis. *International Journal of Molecular Sciences* **2019**, *20*, 2516.
82. Bahirat, U.A.; Shenoy, R.R.; Goel, R.N.; Nemmani, K.V. APD668, a G protein-coupled receptor 119 agonist improves fat tolerance and attenuates fatty liver in high-trans fat diet induced steatohepatitis model in C57BL/6 mice. *European Journal of Pharmacology* **2017**, *801*, 35–45.
83. Sierra, S.; Luquin, N.; Navarro-Otano, J. The endocannabinoid system in cardiovascular function: novel insights and clinical implications. *Clinical Autonomic Research* **2018**, *28*, 35–52.
84. Malinowska, B.; Toczek, M.; Pędzińska-Betiuk, A.; Schlicker, E. Cannabinoids in arterial, pulmonary and portal hypertension–mechanisms of action and potential therapeutic significance. *British Journal of Pharmacology* **2019**, *176*, 1395–1411.
85. Toczek, M.; Baranowska-Kuczko, M.; Grzęda, E.; Pędzińska-Betiuk, A.; Weresa, J.; Malinowska, B. Age-specific influences of chronic administration of the fatty acid amide hydrolase inhibitor URB597 on cardiovascular parameters and organ hypertrophy in DOCA-salt hypertensive rats. *Pharmacological Reports* **2016**, *68*, 363–369.
86. Ahmad, A.; Dempsey, S.K.; Daneva, Z.; Li, N.; Poklis, J.L.; Li, P.L.; Ritter, J.K. Modulation of mean arterial pressure and diuresis by renomedullary infusion of a selective inhibitor of fatty acid amide hydrolase. *American Journal of Physiology-Renal Physiology* **2018**, *315*, F967–F976.
87. Sugamura, K.; Sugiyama, S.; Fujiwara, Y.; Matsubara, J.; Akiyama, E.; Maeda, H.; Ohba, K.; Matsuzawa, Y.; Konishi, M.; Nozaki, T.; others. Cannabinoid 1 receptor blockade reduces atherosclerosis with enhances reverse cholesterol transport. *Journal of Atherosclerosis and Thrombosis* **2010**, *17*, 141–147.
88. Guillamat-Prats, R.; Rami, M.; Herzig, S.; Steffens, S. Endocannabinoid signalling in atherosclerosis and related metabolic complications. *Thrombosis and Haemostasis* **2019**, *119*, 567–575.
89. Prats, R.G.; Rami, M.; Ring, L.; Rinne, P.; Lauer, E.; Lenglet, S.; Thomas, A.; Pagano, S.; Vuilleumier, N.; Cravatt, B.F.; others. Deficiency of monoacylglycerol lipase enhances IgM plasma levels and limits atherogenesis in a CB2-dependent manner. *Thrombosis and Haemostasis* **2019**, *119*, 348–351.
90. Rinne, P.; Guillamat-Prats, R.; Rami, M.; Bindila, L.; Ring, L.; Lyytikäinen, L.P.; Raitoharju, E.; Oksala, N.; Lehtimäki, T.; Weber, C.; others. Palmitoylethanolamide promotes a proresolving macrophage phenotype and attenuates atherosclerotic plaque formation. *Arteriosclerosis, Thrombosis, and Vascular Biology* **2018**, *38*, 2562–2575.
91. Schloss, M.J.; Horckmans, M.; Guillamat-Prats, R.; Hering, D.; Lauer, E.; Lenglet, S.; Weber, C.; Thomas, A.; Steffens, S. 2-Arachidonoylglycerol mobilizes myeloid cells and worsens heart function after acute myocardial infarction. *Cardiovascular Research* **2019**, *115*, 602–613.

92. Duerr, G.D.; Heinemann, J.C.; Kley, J.; Eichhorn, L.; Frede, S.; Weisheit, C.; Wehner, S.; Bindila, L.; Lutz, B.; Zimmer, A.; others. Myocardial maladaptation to pressure overload in CB2 receptor-deficient mice. *Journal of Molecular and Cellular Cardiology* **2019**, *133*, 86–98.
93. Hai, K.; Chen, G.; Gou, X.; Jang, H.; Gong, D.; Cheng, Y.; Gong, C.; Li, X.; Liu, Y.; Li, H.; others. Monoacylglycerol Lipase Inactivation by Using URB602 Mitigates Myocardial Damage in a Rat Model of Cardiac Arrest. *Critical Care Medicine* **2019**, *47*, e144–e151.
94. Khan, M.I.; Sobocińska, A.A.; Brodaczewska, K.K.; Zielniok, K.; Gajewska, M.; Kieda, C.; Czarnecka, A.M.; Szczylik, C. Involvement of the CB 2 cannabinoid receptor in cell growth inhibition and G0/G1 cell cycle arrest via the cannabinoid agonist WIN 55,212–2 in renal cell carcinoma. *BMC Cancer* **2018**, *18*, 583.
95. Youssif, B.G.; Mohamed, A.M.; Osman, E.E.A.; Abou-Ghadir, O.F.; Elnaggar, D.H.; Abdelrahman, M.H.; Treamblu, L.; Gomaa, H.A. 5-Chlorobenzofuran-2-carboxamides: From allosteric CB1 modulators to potential apoptotic antitumor agents. *European Journal of Medicinal Chemistry* **2019**, *177*, 1–11.
96. Velasco, G.; Sánchez, C.; Guzmán, M. Towards the use of cannabinoids as antitumour agents. *Nature Reviews Cancer* **2012**, *12*, 436–444.
97. Kargl, J.; Andersen, L.; Hasenöhrl, C.; Feuersinger, D.; Stančić, A.; Fauland, A.; Magnes, C.; El-Heliebi, A.; Lax, S.; Uranitsch, S.; others. GPR55 promotes migration and adhesion of colon cancer cells indicating a role in metastasis. *British Journal of Pharmacology* **2016**, *173*, 142–154.
98. Andradas, C.; Blasco-Benito, S.; Castillo-Lluva, S.; Dillenburg-Pilla, P.; Diez-Alarcia, R.; Juanes-García, A.; García-Taboada, E.; Hernando-Llorente, R.; Soriano, J.; Hamann, S.; others. Activation of the orphan receptor GPR55 by lysophosphatidylinositol promotes metastasis in triple-negative breast cancer. *Oncotarget* **2016**, *7*, 47565–47575.
99. Hasenoehrl, C.; Feuersinger, D.; Sturm, E.M.; Bärnthaler, T.; Heitzer, E.; Graf, R.; Grill, M.; Pichler, M.; Beck, S.; Butcher, L.; others. G protein-coupled receptor GPR55 promotes colorectal cancer and has opposing effects to cannabinoid receptor 1. *International Journal of Cancer* **2018**, *142*, 121–132.
100. Zhou, X.l.; Guo, X.; Song, Y.p.; Zhu, C.y.; Zou, W. The LPI/GPR55 axis enhances human breast cancer cell migration via HBXIP and p-MLC signaling. *Acta Pharmacologica Sinica* **2018**, *39*, 459–471.
101. Fonseca, B.M.; Correia-da Silva, G.; Teixeira, N. Cannabinoid-induced cell death in endometrial cancer cells: involvement of TRPV1 receptors in apoptosis. *Journal of Physiology and Biochemistry* **2018**, *74*, 261–272.
102. Ramer, R.; Schwarz, R.; Hinz, B. Modulation of the Endocannabinoid System as a Potential Anticancer Strategy. *Frontiers in Pharmacology* **2019**, *10*, 430.
103. Brunetti, L.; Loiodice, F.; Piemontese, L.; Tortorella, P.; Laghezza, A. New Approaches to Cancer Therapy: Combining Fatty Acid Amide Hydrolase (FAAH) Inhibition with Peroxisome Proliferator-Activated Receptors (PPARs) Activation: Miniperspective. *Journal of Medicinal Chemistry* **2019**, *62*, 10995–11003.
104. Ma, M.; Bai, J.; Ling, Y.; Chang, W.; Xie, G.; Li, R.; Wang, G.; Tao, K. Monoacylglycerol lipase inhibitor JZL184 regulates apoptosis and migration of colorectal cancer cells. *Molecular Medicine Reports* **2016**, *13*, 2850–2856.
105. Granchi, C.; Lapillo, M.; Glasmacher, S.; Bononi, G.; Licari, C.; Poli, G.; El Boustani, M.; Caligiuri, I.; Rizzolio, F.; Gertsch, J.; others. Optimization of a benzoylpiperidine class identifies a highly potent and selective reversible monoacylglycerol lipase (MAGL) inhibitor. *Journal of Medicinal Chemistry* **2019**, *62*, 1932–1958.
106. Li, X.; Gao, S.; Li, W.; Liu, Z.; Shi, Z.; Qiu, C.; Jiang, J. Effect of monoacylglycerol lipase on the tumor growth in endometrial cancer. *Journal of Obstetrics and Gynaecology Research* **2019**, *45*, 2043–2054.
107. Marino, S.; de Ridder, D.; Bishop, R.T.; Renema, N.; Ponzetti, M.; Sophocleous, A.; Capulli, M.; Aljeffery, A.; Carrasco, G.; Gens, M.D.; others. Paradoxical effects of JZL184, an inhibitor of monoacylglycerol lipase, on bone remodelling in healthy and cancer-bearing mice. *EBioMedicine* **2019**, *44*, 452–466.
108. Vago, R.; Bettiga, A.; Salonia, A.; Ciuffreda, P.; Ottria, R. Development of new inhibitors for N-acylethanolamine-hydrolyzing acid amidase as promising tool against bladder cancer. *Bioorganic & Medicinal Chemistry* **2017**, *25*, 1242–1249.
109. Ashton, J.C.; Hancox, R.J. The Case for Cannabinoid CB1 Receptors as a Target for Bronchodilator Therapy for β-agonist Resistant Asthma. *Current Drug Targets* **2018**, *19*, 1344–1349.
110. Sharkey, K.A.; Darmani, N.A.; Parker, L.A. Regulation of nausea and vomiting by cannabinoids and the endocannabinoid system. *European Journal of Pharmacology* **2014**, *722*, 134–146.

111. Darmani, N.A.; Chebolu, S.; Zhong, W.; Trinh, C.; McClanahan, B.; Brar, R.S. Additive antiemetic efficacy of low-doses of the cannabinoid CB1/2 receptor agonist Δ9-THC with ultralow-doses of the vanilloid TRPV1 receptor agonist resiniferatoxin in the least shrew (Cryptotis parva). *European Journal of Pharmacology* **2014**, *722*, 147–155.
112. Ambrose, T.; Simmons, A. Cannabis, cannabinoids, and the endocannabinoid system—is there therapeutic potential for inflammatory bowel disease? *Journal of Crohn's and Colitis* **2019**, *13*, 525–535.
113. Grill, M.; Högenauer, C.; Blesl, A.; Haybaeck, J.; Golob-Schwarzl, N.; Ferreirós, N.; Thomas, D.; Gurke, R.; Trötzmüller, M.; Köfeler, H.C.; others. Members of the endocannabinoid system are distinctly regulated in inflammatory bowel disease and colorectal cancer. *Scientific Reports* **2019**, *9*, 2358.
114. Rossi, F.; Tortora, C.; Punzo, F.; Bellini, G.; Argenziano, M.; Di Paola, A.; Torella, M.; Perrotta, S. The Endocannabinoid/Endovanilloid System in Bone: From Osteoporosis to Osteosarcoma. *International Journal of Molecular Sciences* **2019**, *20*, 1919.
115. Jiang, H.; Wu, Y.; Valverde, P.; Murray, D.; Tang, J.; Yao, Q.; Han, Q.; Zhang, J.; Zhang, L.; Sui, L.; others. Central adiponectin induces trabecular bone mass partly through epigenetic downregulation of cannabinoid receptor CB1. *Journal of Cellular Physiology* **2019**, *234*, 7062–7069.
116. Castelli, M.; Piras, A.; Melis, T.; Succu, S.; Sanna, F.; Melis, M.; Collu, S.; Ennas, M.G.; Diaz, G.; Mackie, K.; others. Cannabinoid CB1 receptors in the paraventricular nucleus and central control of penile erection: immunocytochemistry, autoradiography and behavioral studies. *Neuroscience* **2007**, *147*, 197–206.
117. Milando, R.; Friedman, A. Cannabinoids: Potential Role in Inflammatory and Neoplastic Skin Diseases. *American Journal of Clinical Dermatology* **2019**, *20*, 167–180.
118. Tóth, K.F.; Ádám, D.; Bíró, T.; Oláh, A. Cannabinoid Signaling in the Skin: Therapeutic Potential of the "C(ut)annabinoid" System. *Molecules* **2019**, *24*, 918.
119. Zákány, N.; Oláh, A.; Markovics, A.; Takács, E.; Aranyász, A.; Nicolussi, S.; Piscitelli, F.; Allarà, M.; Pór, Á.; Kovács, I.; others. Endocannabinoid Tone Regulates Human Sebocyte Biology. *Journal of Investigative Dermatology* **2018**, *138*, 1699–1706.
120. Iannotti, F.A.; Pagano, E.; Guardiola, O.; Adinolfi, S.; Saccone, V.; Consalvi, S.; Piscitelli, F.; Gazzerro, E.; Busetto, G.; Carrella, D.; others. Genetic and pharmacological regulation of the endocannabinoid CB1 receptor in Duchenne muscular dystrophy. *Nature Communications* **2018**, *9*, 3950.
121. Podda, G.; Constantinescu, C.S. Nabiximols in the treatment of spasticity, pain and urinary symptoms due to multiple sclerosis. *Expert Opinion on Biological Therapy* **2012**, *12*, 1517–1531.
122. May, M.B.; Glode, A.E. Dronabinol for chemotherapy-induced nausea and vomiting unresponsive to antiemetics. *Cancer Management and Research* **2016**, *8*, 49–55.
123. Beal, J.E.; Olson, R.; Laubenstein, L.; Morales, J.O.; Bellman, P.; Yangco, B.; Lefkowitz, L.; Plasse, T.F.; Shepard, K.V. Dronabinol as a treatment for anorexia associated with weight loss in patients with AIDS. *Journal of Pain and Symptom Management* **1995**, *10*, 89–97.
124. Deng, H.; Li, W. Monoacylglycerol Lipase Inhibitors: Modulators for Lipid Metabolism in Cancer Malignancy, Neurological and Metabolic Disorders. *Acta Pharmaceutica Sinica B* **2019**.
125. Lagatta, D.C.; Kuntze, L.B.; Ferreira-Junior, N.C.; Resstel, L.B. Medial prefrontal cortex TRPV1 and CB1 receptors modulate cardiac baroreflex activity by regulating the NMDA receptor/nitric oxide pathway. *Pflügers Archiv-European Journal of Physiology* **2018**, *470*, 1521–1542.
126. Song, S.; Ayon, R.J.; Yamamura, A.; Yamamura, H.; Dash, S.; Babicheva, A.; Tang, H.; Sun, X.; Cordery, A.G.; Khalpey, Z.; others. Capsaicin-induced Ca2+ signaling is enhanced via upregulated TRPV1 channels in pulmonary artery smooth muscle cells from patients with idiopathic PAH. *American Journal of Physiology-Lung Cellular and Molecular Physiology* **2017**, *312*, L309–L325.
